# Disentangling Alzheimer’s disease neurodegeneration from typical brain ageing using machine learning

**DOI:** 10.1093/braincomms/fcac117

**Published:** 2022-05-07

**Authors:** Gyujoon Hwang, Ahmed Abdulkadir, Guray Erus, Mohamad Habes, Raymond Pomponio, Haochang Shou, Jimit Doshi, Elizabeth Mamourian, Tanweer Rashid, Murat Bilgel, Yong Fan, Aristeidis Sotiras, Dhivya Srinivasan, John C. Morris, Marilyn S. Albert, Nick R. Bryan, Susan M. Resnick, Ilya M. Nasrallah, Christos Davatzikos, David A. Wolk, from the iSTAGING consortium, for the ADNI

**Affiliations:** 1 Center for Biomedical Image Computing and Analytics, University of Pennsylvania, Philadelphia, PA, USA; 2 Department of Radiology, University of Pennsylvania, Philadelphia, PA, USA; 3 Glenn Biggs Institute for Alzheimer’s & Neurodegenerative Diseases, University of Texas Health Science Center at San Antonio, San Antonio, TX, USA; 4 Penn Statistics in Imaging and Visualization Center, Department of Biostatistics, Epidemiology, and Informatics, Perelman School of Medicine, University of Pennsylvania, Philadelphia, PA, USA; 5 Laboratory of Behavioral Neuroscience, National Institute on Aging, Baltimore, MD, USA; 6 Department of Radiology, Washington University in St Louis, St Louis, MO, USA; 7 Department of Neurology, Washington University in St Louis, St Louis, MO, USA; 8 Department of Neurology, Johns Hopkins University School of Medicine, Baltimore, MD, USA; 9 Department of Diagnostic Medicine, University of Texas, Austin, TX, USA; 10 Department of Neurology and Penn Memory Center, University of Pennsylvania, Philadelphia, PA, USA

**Keywords:** Alzheimer’s disease, brain ageing, MRI biomarker, amyloid, machine learning

## Abstract

Neuroimaging biomarkers that distinguish between changes due to typical brain ageing and Alzheimer’s disease are valuable for determining how much each contributes to cognitive decline. Supervised machine learning models can derive multivariate patterns of brain change related to the two processes, including the Spatial Patterns of Atrophy for Recognition of Alzheimer’s Disease (SPARE-AD) and of Brain Aging (SPARE-BA) scores investigated herein. However, the substantial overlap between brain regions affected in the two processes confounds measuring them independently. We present a methodology, and associated results, towards disentangling the two.

T_1_-weighted MRI scans of 4054 participants (48–95 years) with Alzheimer’s disease, mild cognitive impairment (MCI), or cognitively normal (CN) diagnoses from the Imaging-based coordinate SysTem for AGIng and NeurodeGenerative diseases (iSTAGING) consortium were analysed. Multiple sets of SPARE scores were investigated, in order to probe imaging signatures of certain clinically or molecularly defined sub-cohorts. First, a subset of clinical Alzheimer’s disease patients (*n* = 718) and age- and sex-matched CN adults (*n* = 718) were selected based purely on clinical diagnoses to train SPARE-BA1 (regression of age using CN individuals) and SPARE-AD1 (classification of CN versus Alzheimer’s disease) models. Second, analogous groups were selected based on clinical and molecular markers to train SPARE-BA2 and SPARE-AD2 models: amyloid-positive Alzheimer’s disease continuum group (*n* = 718; consisting of amyloid-positive Alzheimer’s disease, amyloid-positive MCI, amyloid- and tau-positive CN individuals) and amyloid-negative CN group (*n* = 718). Finally, the combined group of the Alzheimer’s disease continuum and amyloid-negative CN individuals was used to train SPARE-BA3 model, with the intention to estimate brain age regardless of Alzheimer’s disease-related brain changes.

The disentangled SPARE models, SPARE-AD2 and SPARE-BA3, derived brain patterns that were more specific to the two types of brain changes. The correlation between the SPARE-BA Gap (SPARE-BA minus chronological age) and SPARE-AD was significantly reduced after the decoupling (*r* = 0.56–0.06). The correlation of disentangled SPARE-AD was non-inferior to amyloid- and tau-related measurements and to the number of APOE ε4 alleles but was lower to Alzheimer’s disease-related psychometric test scores, suggesting the contribution of advanced brain ageing to the latter. The disentangled SPARE-BA was consistently less correlated with Alzheimer’s disease-related clinical, molecular and genetic variables.

By employing conservative molecular diagnoses and introducing Alzheimer’s disease continuum cases to the SPARE-BA model training, we achieved more dissociable neuroanatomical biomarkers of typical brain ageing and Alzheimer’s disease.

## Teaser

Structural brains that are experiencing accelerated ageing can be misinterpreted to have Alzheimer’s disease and vice versa. Hwang *et al*. develop machine learning-based metrics to measure them more independently and report that the disentangled metrics offer better dimensional insights into how the two are differentially associated with clinical outcomes.

## Graphical Abstract

**Graphical Abstract:**
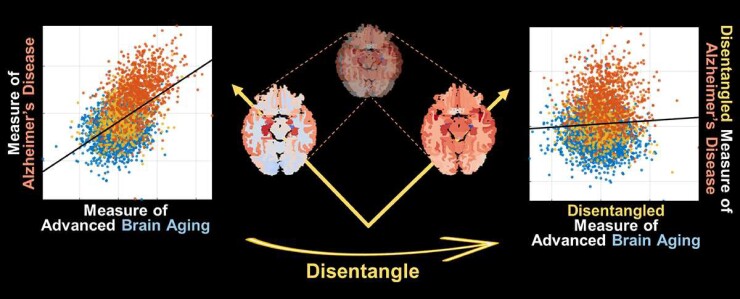

## Introduction

Ageing is a complex process that can be broadly defined as progressive loss of physiological integrity or gradual accumulation of deleterious biological changes accompanying loss of function.^1,2^ While many methods for developing biomarkers of brain ageing have been proposed,^3^ markers based on structural MRI (commonly known as the ‘brain age’) show less inter-individual variability and methodological variations of measurements relative to other modalities.^4,5^ Generally, brain age is computed by training a multi-dimensional regression model with structural brain features (region- or voxel-based) to predict the chronological ages of healthy individuals.^6^ This model then looks for the learned structural patterns in an unseen brain to predict the age. The difference between the predicted brain age and the actual chronological age, often known as the ‘brain age gap’, ‘brain age gap estimation’, ‘brain age delta’, or ‘predicted age difference’, can be used to assess whether the brain looks appropriate for the chronological age or displays deviance from expectation.^7,8^

A similar approach can be employed to find structural patterns for other types of brain changes. For example, a machine learning-based score known as the Spatial Pattern of Atrophy for Recognition of Alzheimer’s Disease (SPARE-AD) captures multivariate brain changes of Alzheimer’s disease and has been extensively validated.^9,10^ It is computed by training a support vector machine (SVM) classification model^11^ to separate between cognitively normal (CN) and Alzheimer’s disease populations using structural brain features and then by measuring the distance of the data points (where each point represents a brain) away from the separating hyperplane.

Summary scores like the SPARE-AD or SPARE-BA, an analogous score for Brain Aging derived using support vector regression (SVR),^12^ provide expressive markers that reflect complex brain changes associated with these conditions. As typical brain ageing processes and neurodegeneration due to Alzheimer’s disease can both affect an individual to varying degrees and account for their cognitive status, one could consider these measures as two axes on a 2D coordinate system where each axis reflects an aspect of structural brain integrity and where each brain can be represented as a point. For example, someone with a similar amount of Alzheimer’s disease pathology may be more or less impaired by the degree to which they experience advanced or normal brain ageing in this context. Information on which direction on the coordinate system a point deviates from the norm may then be useful.

**Figure 1:**
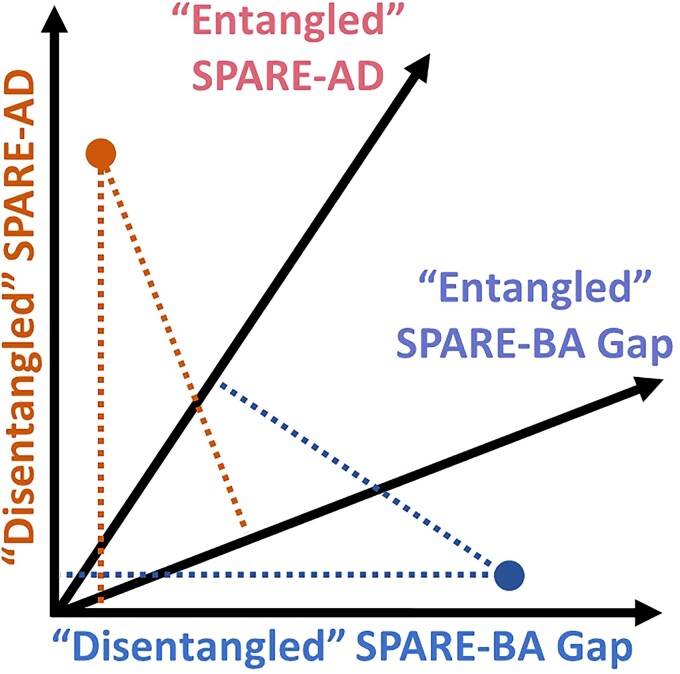
**Hypothetical SPARE scores of two individuals**. The dot on the top left corner represents a person suffering from Alzheimer’s disease, with little advanced brain ageing. The dot on the bottom right corner represents a person suffering from advanced brain ageing, with little Alzheimer’s disease pathology. If the SPARE-AD and SPARE-BA are correlated or ‘entangled’, both individuals would receive elevated SPARE-AD and SPARE-BA Gap (SPARE-BA minus chronological age, capturing ‘advanced’ brain ageing). The two cases would be better differentiated with orthogonalized or ‘disentangled’ SPARE scores.

**Figure 2:**
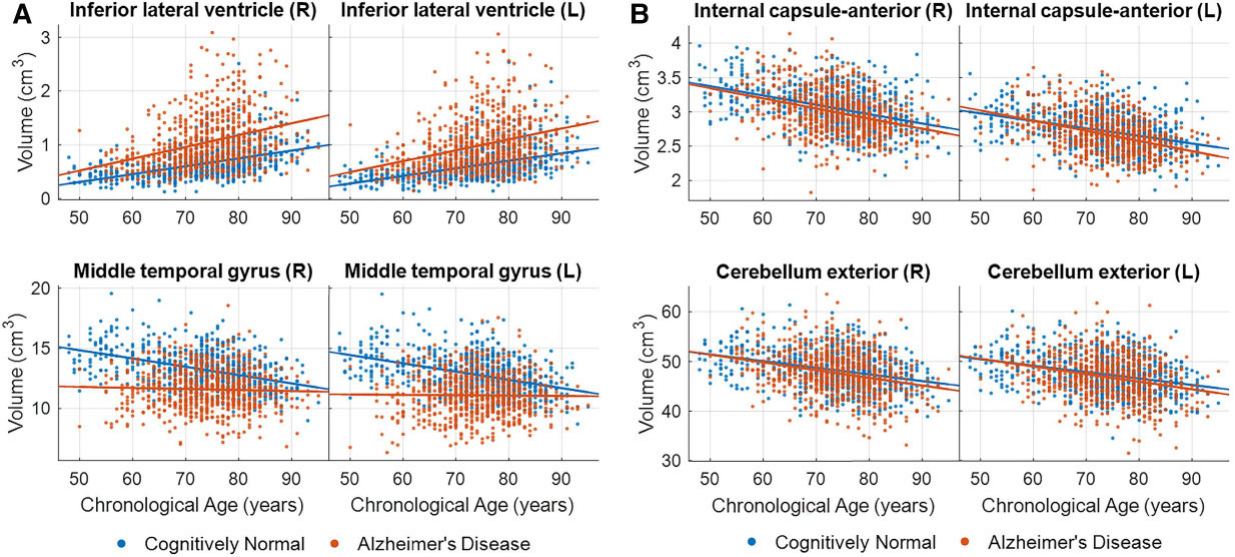
**Examples of brain regions associated with typical ageing**. Some regions are further influenced by Alzheimer’s disease status **(A)**, while others look similar in clinical Alzheimer’s disease patients relative to cognitively normal participants **(B)**. For the SPARE-BA and SPARE-AD scores to be disentangled, we want the SPARE-BA model to be trained with features in **(B)**, which would most likely be ignored by the SPARE-AD model whose objective is to differentiate between the two groups. L, left hemisphere; R, right hemisphere.

One caveat with this approach when it comes to Alzheimer’s disease is that many brain regions that are associated with Alzheimer’s disease are also associated with typical brain ageing,^13,14^ which creates an inherent correlation between the two scores. We refer to it here as an ‘entanglement’ of the multivariate brain patterns captured by the SPARE-BA and SPARE-AD models or simply an ‘entanglement’ of the two corresponding axes (Fig. 1). For example, brain volumes of regions such as inferior lateral ventricles or middle temporal gyri correlate both with age and the presence of Alzheimer’s disease (Fig. 2A). Therefore, volume changes in these regions would similarly affect both SPARE-BA and SPARE-AD scores, resulting in bias and correlation of the two scores. Indeed, there are numerous reports of increased brain age in Alzheimer’s disease patients^4,5,15,16^ and increased SPARE-AD with age in CN individuals,^9,10,17^ but to our knowledge, this is the first work to systematically address these confounds.

Another potential contributor to this unwanted correlation between the two SPARE scores is the low sensitivity and specificity of the diagnosis of Alzheimer’s disease.^18^ The low sensitivity results in individuals with asymptomatic Alzheimer’s disease pathology grouped together with CN individuals when training the SPARE-BA model.^19^ As the proportion of CN individuals with Alzheimer’s disease pathology increases with age, this confound is likely to be more pronounced in the older age range.^20^ In this regard, a recent publication suggested that restricting the training sample of a brain age model to only amyloid-negative CN individuals could improve its performance as a biomarker.^21^ On the other hand, low specificity results in cognitively impaired individuals with non-Alzheimer’s disease pathologies being included in the Alzheimer’s disease group,^22^ reducing the ability of the SPARE-AD model to detect Alzheimer’s disease-specific brain changes. We hypothesized that a careful selection of the training samples using molecular biomarkers specific to Alzheimer’s disease^23–25^ may reduce the correlation between the two SPARE scores.

While Alzheimer’s disease and brain ageing do display overlap in regions of atrophy, there are also reported differences in the two structural patterns.^13,14^ Thus, if we can train the SPARE-BA model such that it learns patterns from outside of the Alzheimer’s disease-related brain regions (such as regions in Fig. 2B, instead of in Fig. 2A), the correlation may be reduced. For example, instead of training the SPARE-BA model using only cognitive normal individuals, which is the usual procedure, we can include individuals with Alzheimer’s disease. We hypothesized that this heterogeneous presence of Alzheimer’s disease across the sample would increase variability in Alzheimer’s disease-related brain region volumes, reducing the strength of their age correlations and making these regions less useful in the age model.

If the axes of SPARE-BA and SPARE-AD can be realigned so that they are least affected by the counterpart, they would construct a better coordinate system where each dimension captures more unique patterns of neurodegeneration. The two axes would together be able to detect individuals with Alzheimer’s disease but with an otherwise typical brain ageing and individuals with advanced brain ageing, presumably due to other pathologies, but without Alzheimer’s disease. When the two measures are entangled, clinical interpretation of both is more challenging. Thus, a disentangled understanding of an ageing brain using these measures would aid clinicians in understanding the severity of Alzheimer’s disease-specific neurodegeneration and in potentially detecting comorbidities if they can be captured by the disentangled SPARE-BA. It would also be helpful in research settings for monitoring outcomes of therapies directed specifically at either source of neurodegeneration.

Therefore, the goal of the present study was to derive more disentangled SPARE-BA and SPARE-AD scores by testing the following two approaches: (i) refine the training samples for both machine learning models by implementing strict molecular diagnostic criteria using amyloid and tau measurements and (ii) add Alzheimer’s disease brains in the training of the SPARE-BA model, which would lead the model to learn ageing patterns with brain features least affected by Alzheimer’s disease. Next, the disentangled SPARE scores were evaluated based on their correlations with demographic, clinical, molecular and genetic variables related to Alzheimer’s disease.

## Materials and methods

### iSTAGING consortium

Imaging-based coordinate SysTem for AGing and NeurodeGenerative diseases (iSTAGING) is a collection of multi-modal neuroimaging data from more than 10 major studies, initiated with the goal of finding reliable and generalizable coordinate systems to capture human brain heterogeneity across a wide range of the age spectrum.^26^ Individuals from four studies in the iSTAGING consortium with amyloid status data were investigated: the Alzheimer’s Disease Neuroimaging Initiative (ADNI; *n* = 1767),^27,28^ Biomarkers of Cognitive Decline Among Normal Individuals (BIOCARD; *n* = 279),^29^ the Baltimore Longitudinal Study of Aging (BLSA; *n* = 980),^30^ and the Open Access Series of Imaging Studies (OASIS; *n* = 1028).^31,32^ Only participants 48 years or older were included, as the youngest participant with Alzheimer’s disease was 48 years old. Only participants who were classified as CN, mild cognitive impairment (MCI), or Alzheimer’s disease from the originating studies were included in the current analysis. The clinical diagnostic criteria were broadly similar across the studies and were described in Supplementary Table 1.

The supervisory committee of each study approved its inclusion in this analysis, and this project was approved by the institutional review board of the University of Pennsylvania. All participants gave written informed consent to each study for data acquisition and analyses according to the Declaration of Helsinki. More detail on each of the four studies can be found in Supplementary Methods 1.

### Image preprocessing and harmonization

A fully automated processing pipeline was applied to each T_1_-weighted scan. It involved correction of magnetic field intensity inhomogeneity^33^ and multi-atlas skull-stripping using study-specific atlases.^34^ Then, 145 brain regions of interest (ROIs) consisting of grey matter, white matter and ventricular CSF were extracted for each scan with a multi-atlas non-linear region segmentation method known as MUSE (MUlti-atlas region Segmentation utilizing Ensembles), which has been validated for its robustness to inter-scanner variabilities.^35,36^ Names of the ROIs are listed in Supplementary Table 2.

Significant study-wise effects are inevitable when pooling a diverse dataset due to a lack of standardization in scan acquisition protocols and scanner types.^37^ Differences in sample demographics, as well as batch effects, also confound the analyses. Therefore, a systematic quality control and harmonization of the ROIs were performed.^38^ This harmonization adjusted the ROI volumes for study-wise differences while preserving non-linear trends related to age and sex learned from CN individuals only. This procedure is based on the methods described in detail by Pomponio *et al*.^38^ and has been validated in another work.^26^ After the study-wise effects were removed, sex-wise differences were corrected per ROI volume, based on CN individuals. For cross-validation, the harmonization was performed first on the training sample, then separately on the testing sample by combining the two samples to prevent information leakage.

### Research design and sample selection

Three different versions of SPARE-BA and SPARE-AD models were designed and were differentiated by suffixes, with each version designed to offer SPARE scores that are more disentangled from the counterpart compared with the preceding version. All versions of SPARE-BA (-BA1, -BA2, -BA3) scores were derived using single linear SVR models, differing only in the training samples. A SPARE-BA score is essentially an age predicted by the SVR model using structural brain features (same as ‘brain age’ in the literature).^7^ Likewise, SPARE-AD1 and SPARE-AD2 scores were derived using single linear SVM classification models, differing only in the training samples.^10^ Only one scan per participant was used as a training sample per model.

For SPARE-AD1 and SPARE-BA1, participants were screened into two groups using diagnostic criteria from the originating studies based solely on their clinical symptoms: those who were labelled as having Alzheimer’s disease (‘Clinical Alzheimer’s disease’ group; *n* = 718), and those labelled as being ‘CN’ group (*n* = 718). Therefore, the SPARE-BA1 regression model was trained with the CN group, and the SPARE-AD1 classification model was trained to separate between the CN and Clinical Alzheimer’s disease groups.

For SPARE-AD2 and SPARE-BA2, participants were screened into two groups using conservative amyloid and tau cutoffs. CSF β-amyloid 1–42 (Aβ42; provided by ADNI and BIOCARD),^39,40^ Pittsburgh compound B ([^11^C]PiB; provided by ADNI, BLSA and OASIS),^41–43^ and [^18^F]florbetapir (also known as [^18^F]AV45; provided by ADNI and OASIS)^44^ were used as measures of amyloid, and CSF total tau (provided by ADNI and BIOCARD)^39^ was used as a measure of tau. CSF total phosphorylated tau (pTau; provided by ADNI and BIOCARD)^45^ was also available but was preserved for validation of the results. Conservative cutoffs were used to better assure ‘positive’ and ‘negative’ status, and the methods to define them for each measure per study are detailed in Supplementary Methods 2. The ‘Alzheimer’s disease Continuum’ group included participants with amyloid-positive (A+) results, regardless of their clinical diagnoses: cognitively normal, MCI, or Alzheimer’s disease. However, the A+ cognitively normal participants were further required to have an elevated CSF total tau (T+) to increase the likelihood that these individuals were displaying downstream pathology of Alzheimer’s disease and neurodegeneration. Therefore, the Alzheimer’s disease Continuum group (*n* = 718 in total) consisted of A+ Alzheimer’s disease (*n* = 290), A+ MCI (*n* = 387) and A+T+ cognitively normal (*n* = 41) individuals. The control group included cognitively normal participants who were amyloid negative (‘A−/CN’ group; *n* = 718). Therefore, the SPARE-BA2 model was trained with the A−/CN group, and the SPARE-AD2 model was trained to separate between the A−/CN and Alzheimer’s disease Continuum groups. To minimize biases, the four groups—CN, Clinical Alzheimer’s disease, A−/CN and Alzheimer’s disease Continuum—were statistically matched for the sample size, mean age and sex ratio (*P* > 0.2) (Table 1).

**Table 1:** Demographic summary of all samples

	By clinical diagnosis		By molecular diagnosis	
Groups	Clinical Alzheimer’s disease (*n* = 718)	CN (*n* = 718)	Alzheimer’s disease Continuum (*n* = 718)	A−/CN (*n* = 718)
*n*				
Clinical diagnosis (Alzheimer’s disease/MCI/CN)	718/0/0	0/0/718	290/387/41	0/0/718
Molecular diagnosis (A+/A−/unclear/no data)	359/14/11/334	47/124/101/446	718/0/0/0	0/718/0/0
Study (ADNI/BIOCARD/BLSA/OASIS)	476/1/17/224	195/135/194/194	621/18/15/64	250/100/118/250
*Demographics*				
Age (years) (mean ± SD/range)	74.1 ± 7.6/50–95	73.5 ± 9.3/48–95	73.6 ± 7.6/48–94	73.0 ± 8.8/49–95
Sex (male/female)	389/329	367/351	389/329	367/351
*Clinical*				
MMSE [mean ± SD (*n*)]	23.1 ± 3.5 (619)	29.0 ± 1.2 (582)	25.8 ± 3.5 (649)	29.0 ± 1.3 (470)
*Amyloid*				
CSF β-amyloid 42^a^ [range (*n*)]	78–275 (128)	37–268 (98)	37–178^b^ (238)	200^b^–305 (173)
[^11^C]PiB SUVR^a^ [range (*n*)]	0.71–5.42 (77)	0.92–3.97 (79)	1.54^b^–5.42 (96)	0.50–1.25^b^ (288)
[^18^F]Florbetapir SUVR [range (*n*)]	0.81–3.41 (219)	0.71–2.67 (118)	1.15^b^–3.41 (427)	0.55–1.05^b^ (310)

SD, standard deviation.

^a^ These measurements were harmonized (see Supplementary Methods 1).

^b^ These numbers were thresholded.

During the sample selection, if multiple scans were available per participant, the last scan with the CN label (or A−/CN), or the first scan with the Clinical Alzheimer’s disease label (or Alzheimer’s disease Continuum) was selected. This procedure was done (i) to achieve better age matching between the control and the affected groups and (ii) to tighten the SVM separating boundaries by rendering more difficult classification tasks (introducing more borderline cases that serve as ‘support vectors’).^11^

SPARE-BA3 model (SVR) was trained with the combined group of A−/CN and Alzheimer’s disease Continuum (*n* = 1436). As discussed earlier, we hypothesized that introducing Alzheimer’s disease cases to the training set would lead to a model that captures brain changes that are least affected by Alzheimer’s disease, thereby reducing the correlation between the two SPARE scores. Then, assuming that SPARE-BA3 was a measure of brain age that was as little associated with Alzheimer’s disease pathology as possible, SPARE-AD3 score was computed by regressing out SPARE-BA3 from SPARE-AD2 score to further reduce the influence of brain ageing on the latter. A flowchart depicting the entire sample selection process is found in Supplementary Fig. 1.

### Model training and testing

All SPARE models were trained using a 10-fold cross-validation. The 145 ROI volumes whose study- and sex-wise differences had been harmonized were normalized to *z*-scores within each fold, based on the control group. Details on the model training and hyperparameter settings can be found in Supplementary Method 3. After the cross-validation, SPARE scores for scans of participants who did not enter the training were estimated by applying all 10 cross-validated models and averaging the scores.

The model fit was assessed using root-mean-square error (RMSE), mean absolute error (MAE) and *R*^2^. For the SPARE-AD classification models, the area-under-the-curve (AUC) and classification accuracy were used. Finally, SPARE-BA3 scores were linearly regressed out from the SPARE-AD2 scores using a 10-fold cross-validation for the creation of SPARE-AD3.

### Disentangling the SPARE scores

Whether the SPARE-BA and SPARE-AD scores were disentangled was assessed in three steps. First, the correlation between the SPARE-BA Gap (SPARE-BA minus chronological age) and SPARE-AD was computed using all participants (*n* = 4054). If multiple scans were available per participant, then only one was selected randomly, while favouring scans that were labelled clinically as Alzheimer’s disease over MCI over cognitively normal. Decreased correlation of these two SPARE scores was considered as evidence of disentanglement. Second, the ability of the SPARE-BA Gap and SPARE-AD to separate between CN and Clinical Alzheimer’s disease and between A−/CN and Alzheimer’s disease Continuum was tested. We hypothesized that the disentanglement would significantly decrease this separability using SPARE-BA Gap as it would be less sensitive to Alzheimer’s disease-related changes, which was the desired goal.

Third, the weights assigned to the 145 brain ROIs by each machine learning model as well as the correlations between the brain volumes and the SPARE scores were compared between models to assess how much of the patterns found by the two SPARE models overlapped. To examine the statistical significance of the weights, a permutation test was performed by retraining each version of the model 5000 times with the labels shuffled (chronological ages in the SVR models, and the group memberships in the SVM models). The null distribution of the weights was compared with the 10 weights from the cross-validation per ROI using two-tailed *t*-tests.^46^

### Clinical, molecular and genetic associations

The correlations between the SPARE scores and the molecular measures were computed. In addition to the amyloid and tau measures used for diagnosis, CSF total pTau was examined. Since there existed large study-wise differences in measurements, only one study with the largest sample was analysed per variable. To examine more localized tau pathology, tau PET measurements, available only in a subset of participants in ADNI,^47^ from entorhinal area and inferior temporal gyrus were also correlated with the SPARE scores.

We also computed correlations between the SPARE scores and clinical test scores related to Alzheimer’s disease, which included Mini-Mental State Examination (MMSE),^48^ Montreal Cognitive Assessment (MoCA),^49^ Alzheimer’s Disease Assessment Scale-Cognitive Subscale (ADAS-Cog),^50,51^ Logical Memory,^52^ and Trail Making Test.^53^ To examine whether the disentangled SPARE-BA contributed to the variance in these scores in patients beyond SPARE-Alzheimer’s disease, nested multivariate linear regression models were trained, first with SPARE-BA3, SPARE-BA3 Gap and SPARE-AD3 as predictors, and subsequently with excluding one predictor at a time. The correlations between the SPARE scores and the number of APOE ε4 alleles^54^ were also computed.

### Statistical analysis

Two-sample *t*-tests were used to examine if the mean age was matched between the four groups—CN, Clinical Alzheimer’s disease, A−/CN and Alzheimer’s disease Continuum (raw *P* > 0.2). *χ*^2^ tests were used to examine if the sex ratio was matched between the groups (raw *P* > 0.2).

All *P*-values were adjusted for multiple comparisons using Benjamini–Hochberg correction when establishing significance. Pearson correlation was used to compute correlations between the SPARE scores. Spearman correlation was used to compute correlations between the SPARE scores and clinical, molecular, or genetic variables, as some variables were non-normally distributed (e.g. MMSE score with the cap at 30) or discretized (e.g. number of APOE ε4 alleles). When checking for changes in correlation between SPARE-BA Gap and SPARE-AD, the significance of difference between the two coefficients was determined with the *F*-statistic computed on Fisher *z*-transformed coefficients. When comparing two nested models, the likelihood ratio test^55^ was used to assess the goodness of fit.

### Data availability

Raw MRI scans as well as all demographic, clinical and genetic information used in this analysis can be obtained from databases of each of the four studies with a reasonable request upon approval. Population-based results from the iSTAGING consortium including the SPARE models are projected to be available in 2022.

## Results

### Molecular diagnosis

1810 out of 3257 (56%) scans labelled as cognitively normal and with amyloid measurements passed our conservative amyloid cutoffs to be included in the A−/CN group. 688 out of 814 (85%) scans labelled as Alzheimer’s disease and with amyloid measurements, 926 out of 1798 (52%) scans labelled as MCI, and 78 out of 3257 (2%) scans labelled as cognitively normal filled our amyloid and tau criteria to be included in the Alzheimer’s disease Continuum group. Table 1 summarizes demographics of the four groups—CN, Clinical Alzheimer’s disease (for training SPARE-BA1 and SPARE-AD1 models), A−/CN and Alzheimer’s disease Continuum (for training subsequent SPARE models).

### Disentangling the SPARE scores

Machine learning cross-validation results are summarized in Supplementary Table 3. Overall, SPARE-BA2 showed the best fit (MAE = 5.18, *R*^2^ = 0.650) among the three versions of SPARE-BA, as the training sample was the most homogeneous. When computed on a common test dataset, age correlation of the SPARE-BA3 was better than that of the SPARE-BA1, both for cognitively normal individuals (MAE = 5.80–6.03, *n* = 1218) and for individuals with either MCI or Alzheimer’s disease (MAE = 5.88–7.68, *n* = 506) (Supplementary Fig. 2). SPARE-AD1 showed better cross-validation results (AUC = 0.89) than SPARE-AD2 (AUC = 0.82) as the task of separating between CN and Clinical Alzheimer’s disease groups using brain volumes was relatively easier compared with separating between A−/CN and Alzheimer’s disease Continuum.

**Figure 3:**
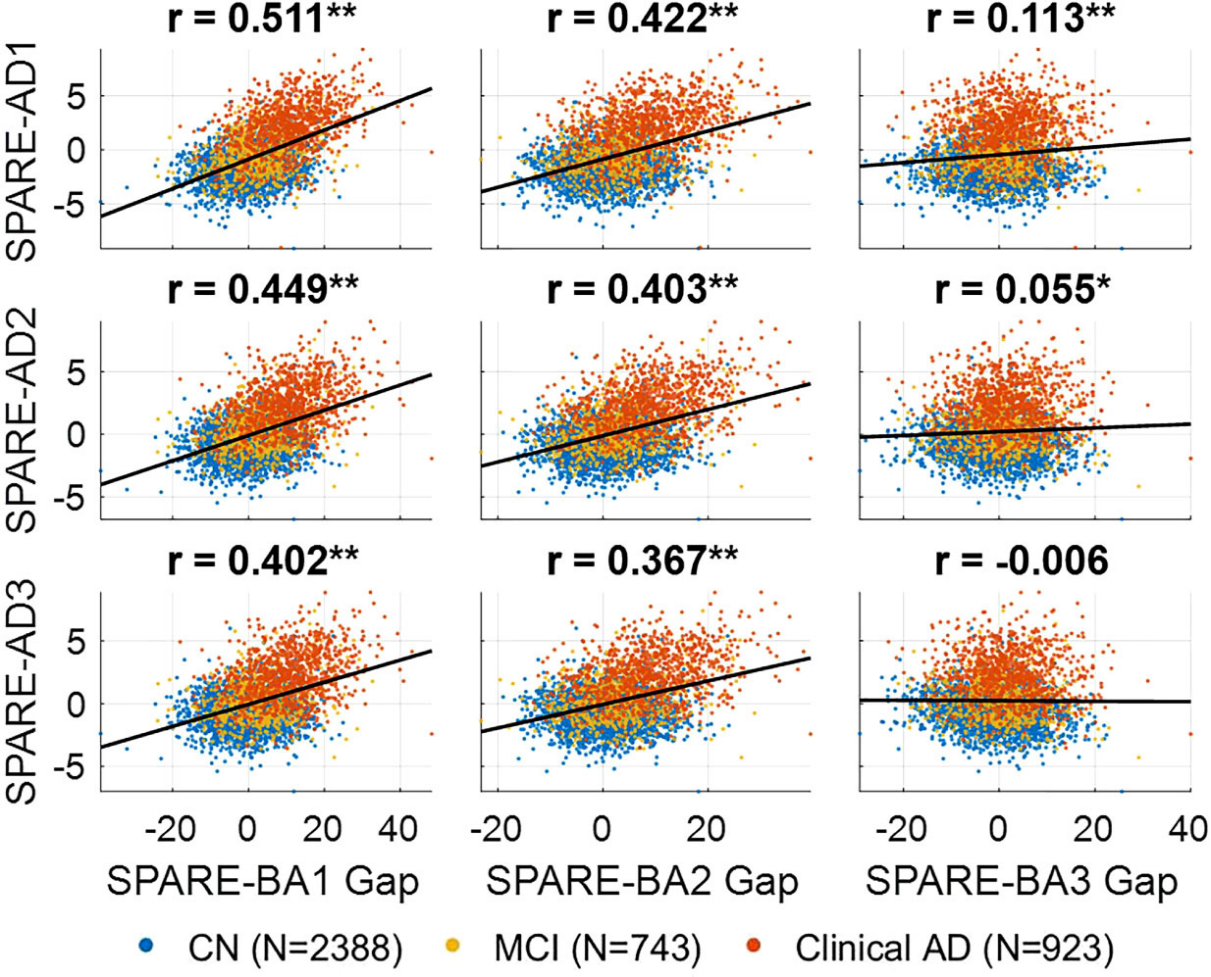
**Correlations between SPARE-BA Gap and SPARE-AD**. Decreased correlation between SPARE-BA Gap (SPARE-BA minus chronological age) and SPARE-AD was considered as evidence of disentanglement. Pearson correlation coefficients including all data points (*n* = 4054) are labelled on top of each subplot. CN, cognitively normal; MCI, mild cognitive impairment; AD, Alzheimer’s disease; *P < 0.05; **P < 0.0001.

However, note that the goal here was not to achieve best fit results, but to build models that produce disentangled SPARE scores. The correlation between SPARE-BA Gap and SPARE-AD was the highest between SPARE-BA1 and SPARE-AD1 (*r* = 0.511, *n* = 4054), which was significantly reduced between SPARE-BA2 and SPARE-AD2 (*r* = 0.403, difference *P*_FDR_ < 0.001), and again between SPARE-BA3 and SPARE-AD2 (*r* = 0.055, difference *P*_FDR_ < 0.001) (Fig. 3). In cognitively normal individuals, SPARE-AD3 was also significantly less correlated with chronological age (*r* = 0.112, *n* = 2388) compared with SPARE-AD2 (*r* = 0.225, difference *P*_FDR_ < 0.001), whose correlation was less compared with SPARE-AD1 (*r* = 0.328, difference *P*_FDR_ < 0.001).

**Figure 4:**
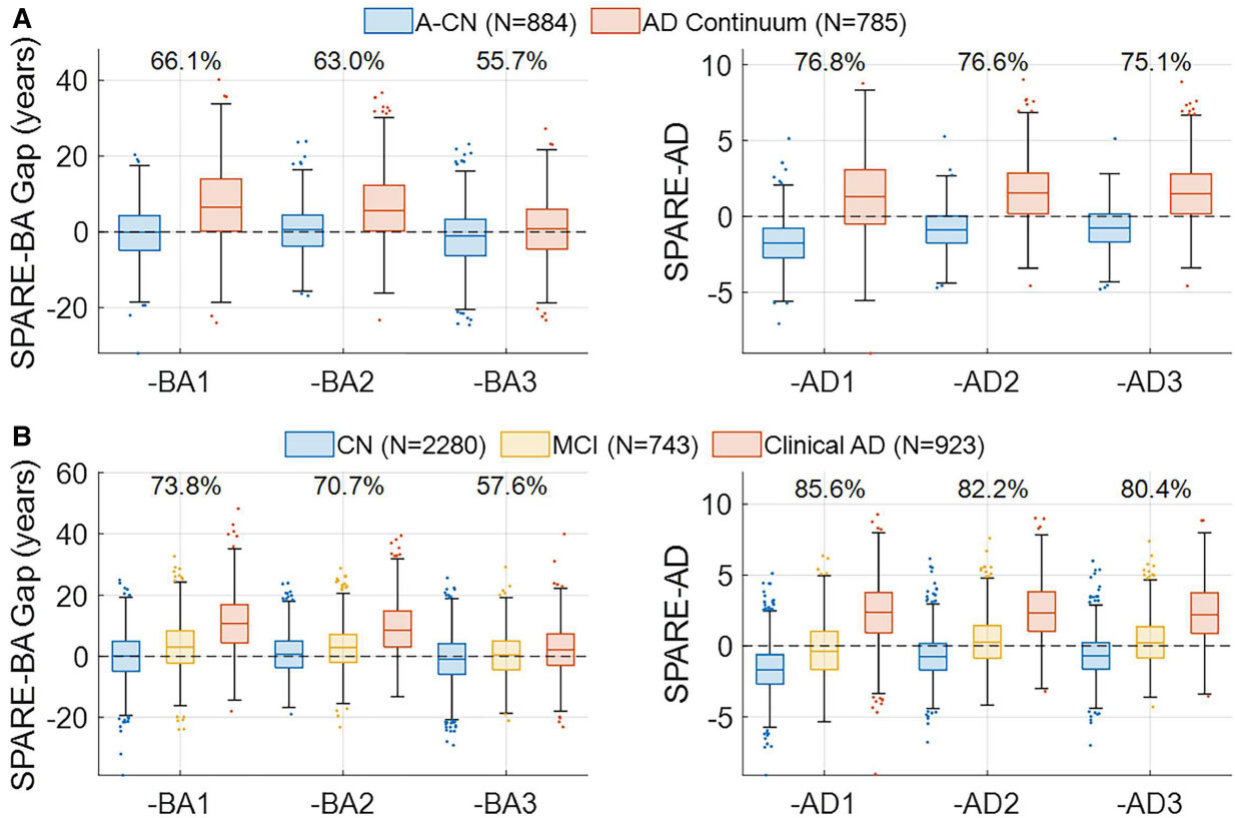
**Distributions of SPARE scores by diagnoses**. Numbers above the boxes are the classification accuracies at **(A)** separating between A−CN and Alzheimer’s disease Continuum (combined group of A+ Alzheimer’s disease, A+ mild cognitive impairment and A+T+ cognitively normal) and **(B)** separating between CN and Clinical Alzheimer’s disease, where sensitivity equals specificity. With SPARE-BA3, the separation is greatly reduced (Cohen’s *d* = 0.30 and 0.43, respectively) compared with SPARE-BA1 (*d* = 0.88 and 1.33, respectively), while the effect sizes of SPARE-AD remain high (*d* > 1.37, d > 1.86, respectively). CN, cognitively normal; MCI, mild cognitive impairment; AD, Alzheimer’s disease.

The classification accuracy of the SPARE-BA Gap at separating between CN and Clinical Alzheimer’s disease was gradually reduced from 73.8% (Cohen’s *d* = 1.33) with SPARE-BA1 to 70.7% (*d* = 1.15) with SPARE-BA2, then to 57.6% (*d* = 0.43) with SPARE-BA3. A similar trend was found with its separability between A−/CN and Alzheimer’s disease Continuum, suggesting progressively greater specificity to ageing unrelated to Alzheimer’s disease. Alternatively, SPARE-AD remained robust at classifying between CN and Clinical Alzheimer’s disease (>80%, *d* > 1.86) and between A−/CN and Alzheimer’s disease Continuum (>75%, *d* > 1.37) (Fig. 4).

### Disentangled regional atrophy patterns

**Figure 5:**
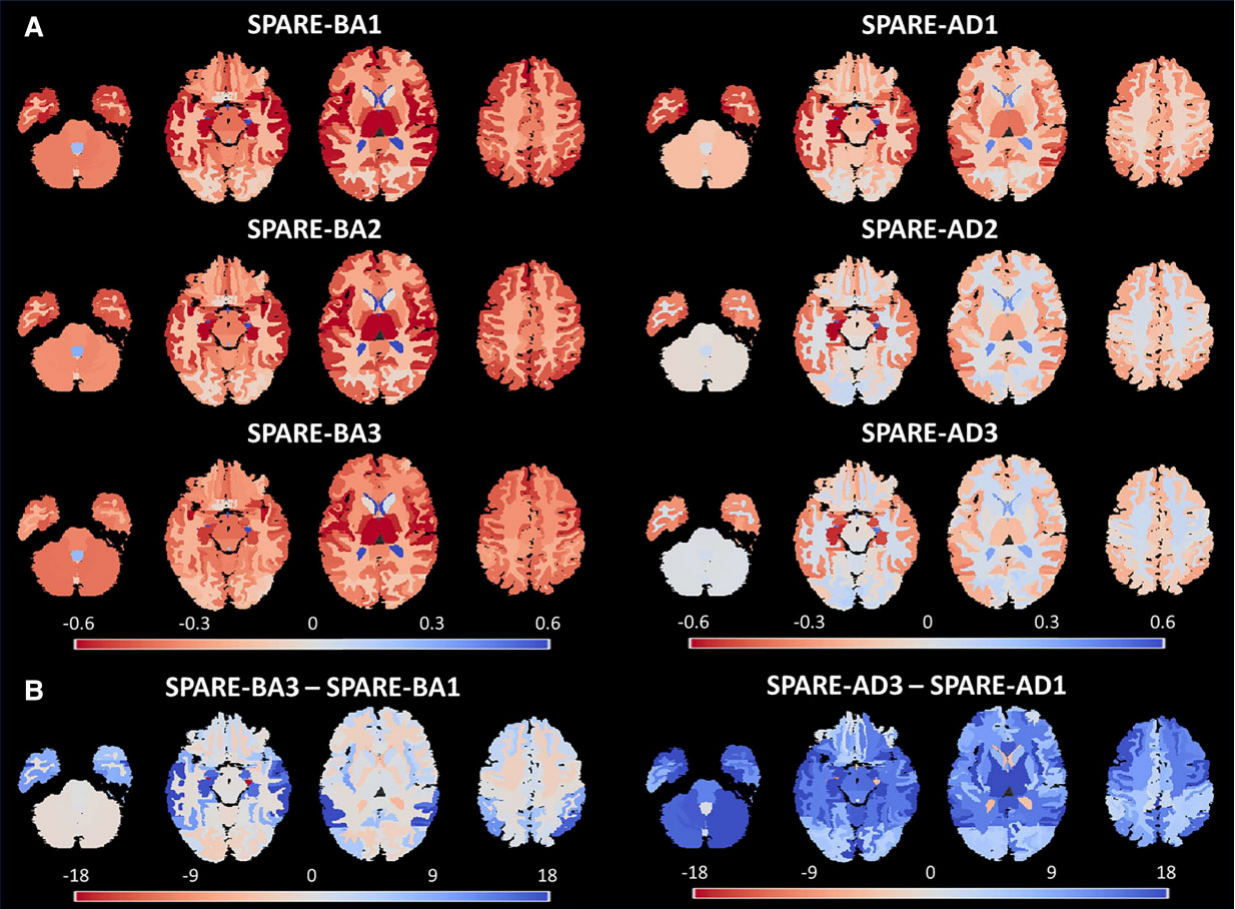
**Brain volumes correlated with the SPARE scores**. (**A**) The colour maps are based on the Spearman correlation rho between the SPARE scores and the brain volumes (*n* = 4054). A negative value indicates a decrease in volume associated with the positive case (older age in SPARE-BA models, and Alzheimer’s disease in SPARE-AD models). (**B**) The colour maps are based on the significance (-log_10_ of the *P*-values) of the correlation changes.

The brain ROIs were evaluated based on both their correlations with the SPARE scores (Fig. 5) and the significance of the assigned weights by the SPARE models (Supplementary Fig. 3). Individual feature weights in a highly multivariate model are heavily influenced by interactions between the features, and therefore, should be interpreted together with the actual correlations between the predictors and predicted response variable (Supplementary Table 4). Cerebellum and anterior limb of internal capsule were among the regions that received more significant weights by the SPARE-BA3 model (*P*_FDR_ < 1E−10) and were more correlated to SPARE-BA3 score compared with the preceding versions (difference *P*_FDR_ < 0.05), highlighting ROIs specific to brain ageing. Regions including inferior lateral ventricles and planum polare showed the opposite trends and became significantly less associated to SPARE-BA3. Most ROIs individually became less correlated to SPARE-AD3 compared with the preceding versions, with hippocampus, middle temporal gyrus, anterior insula, entorhinal area being the most significant (difference *P*_FDR_ < 1E−25), suggesting that these regions were also associated with typical brain ageing. Compared with SPARE-BA2, the SPARE-BA3 model consistently assigned greater weights to brain regions that were least affected by Alzheimer’s disease and smaller weights to the affected regions (Supplementary Fig. 4).

### Clinical, molecular and genetic associations

Consistent with greater specificity to ageing, SPARE-BA3 did not significantly correlate with any tau measures in individuals with either MCI or Alzheimer’s disease. Further, it was significantly less correlated with amyloid and tau measures, compared with SPARE-BA2 and SPARE-BA1 (Table 2 and Supplementary Table 5). SPARE-AD2 correlated more strongly with Aβ42 (*ρ* = −0.400, *n* = 773) and [^18^F]florbetapir (*ρ* = 0.396, *n* = 759) compared with SPARE-AD1 without reaching statistical significance.

**Table 2:** Spearman correlations between SPARE scores and Alzheimer’s disease-related variables

Variable	*n*	Age	SPARE-BA1	SPARE-BA2	SPARE-BA3	SPARE-AD1	SPARE-AD2	SPARE-AD3
*Molecular (amyloid)*								
CSF Aβ42	773	−0.095*	−0.248**	−0.250**	−0.076^*,†^	−0.387**	−**0**.400**	−0.399**
[^18^F]Florbetapir (AV45) SUVR	759	0.101*	0.255**	0.248**	0.100^*,†^	0.384**	**0**.396**	0.396**
*Molecular (tau)*								
CSF total tau	760	0.132*	0.239**	0.219**	0.064^†^	**0**.425**	0.397**	0.397**
CSF total phosphorylated tau	773	0.018	0.116*	0.105*	−0.040^†^	0.302**	0.308**	**0**.313**
Tau PET (entorhinal area)	258	0.008	0.234*	0.191*	0.031^†^	**0**.477**	0.430**	0.428**
Tau PET (inferior temporal gyrus)	258	−0.069	0.184*	0.141*	−0.014	0.402**	0.410**	**0**.415**
*Psychometric*								
MMSE	1310	−0.184**	−0.503**	−0.487**	−0.269^**,†^	−**0**.609**	−0.557^**,†^	−0.540**
MOCA	836	−0.229**	−0.493**	−0.477**	−0.286^**,†^	−**0**.566**	−0.518**	−0.497**
ADAS-Cog 13	1306	0.185**	0.512**	0.499**	0.259^**,†^	**0**.668**	0.624**	0.607**
Logical memory (delayed)	1244	−0.124**	−0.422**	−0.402**	−0.214^**,†^	−**0**.594**	−0.546**	−0.533**
Trail Making Test (Part A)	1215	0.172**	0.401**	**0**.403**	0.243^**,†^	0.368**	0.348**	0.330**
*Genetic*								
APOE4 alleles^a^	1753	−0.121**	0.102**	0.095*	−0.031^†^	0.296**	0.293**	**0**.300**

Correlations in individuals with either mild cognitive impairment or Alzheimer’s disease are shown. More comprehensive table can be found in Supplementary Table 3. Highest correlations per variable are highlighted.

* Corrected *P* < 0.05.

** Corrected *P* < 0.0001.

^†^ Significant difference from the value on the left (*P* < 0.05).

^a^ Both patients and cognitively normal individuals.

SPARE-AD2 and SPARE-AD3 (*ρ* = 0.313, *n* = 773) were more correlated with CSF total phosphorylated tau compared with SPARE-AD1 (*ρ* = 0.302), while the opposite pattern was observed for CSF total tau (*ρ* = 0.425 with SPARE-AD1 and *ρ* = 0.397 with SPARE-AD3, *n* = 760). SPARE-AD1 was the strongest predictor of tau PET standard uptake value ratio (SUVR) in entorhinal area (*ρ* = 0.477, *n* = 258), whereas SPARE-AD3 was the strongest predictor of tau PET SUVR in inferior temporal gyrus (*ρ* = 0.415, *n* = 258). When considering patients and cognitively normal individuals together, SPARE-AD3 was more correlated with the number of APOE ε4 alleles (*ρ* = 0.300, *n* = 1753) compared with SPARE-AD2 and SPARE-AD1 (*ρ* = 0.296) (Table 2). Overall, differences in correlations of SPARE-AD scores with molecular measures and APOE ε4 were not statistically significant, supporting the notion that the changes in approach, including regressing out brain age in SPARE-AD3, did not diminish its specificity to Alzheimer’s disease. In the context of SPARE-BA3 not displaying correlation with these molecular measures, these findings support our success in disentanglement to the underlying biology of Alzheimer’s disease.

SPARE-BA3 was also significantly less correlated with all Alzheimer’s disease-related clinical test scores examined herein compared with SPARE-BA2 and SPARE-BA1 (difference *P*_FDR_ < 0.05) (Table 2). Nonetheless, its correlations with all test scores remained significant, and either SPARE-BA3 or SPARE-BA3 Gap was still a significant contributor to the multivariate models predicting the test scores according to the likelihood ratio test (Supplementary Table 6), suggesting that it provided additional explanatory value beyond SPARE-AD3 even when decoupled from Alzheimer’s disease-related brain changes. This supports the notion that brain ageing itself contributes to varying degrees to the cognitive phenotype of patients with Alzheimer’s disease.

## Discussion

Developing reliable and expressive metrics that summarize complex, high-dimensional brain patterns are critical in neuroimaging. The SPARE scores are designed to capture patterns of specific brain changes to serve as summary metrics. However, given overlap in affected brain regions, structural brain-based measures of typical brain ageing and Alzheimer’s disease are expected to correlate. Here, we show that this correlation can be effectively eliminated by carefully restricting the training samples and by introducing abnormal cases to the brain ageing models.

### Molecular definition of Alzheimer’s disease

With the advancements in both biofluid and imaging-based biomarkers, the field has been moving towards a more biologically based definition of Alzheimer’s disease.^24,56^ This has recently been formulated in the National Institute of Aging-Alzheimer’s Association research framework,^57^ in which patients are dichotomously classified along three dimensions: amyloid (A), tau (T) and neurodegeneration (N).^58^ Parallel to neuropathological definitions of Alzheimer’s disease, one must have the presence of both amyloid and tau biomarkers (A+, T+) to be classified as having Alzheimer’s disease. These efforts reflect the fact that diagnosis of Alzheimer’s disease based solely on cognitive symptoms is not sufficient and may cause significant false positive and false negative results.^18^

In this context, we tested whether defining training samples based on biological markers such as amyloid and tau can improve the specificity of the structural brain imaging markers. As any measurement would inherently contain variance, we excluded individuals at the borderline by using stricter cutoffs than what are commonly used. Only 1810 out of 3257 (56%) scans from individuals who were labelled as cognitively normal passed our conservative cutoff for being A−, while 688 out of 814 (85%) scans from individuals that were labelled clinically as Alzheimer’s disease passed the cutoff for being A+. This supports the heterogeneity that exists in samples that are solely defined by clinical symptoms, and as discussed earlier, misclassified individuals result in measures of brain ageing and Alzheimer’s disease that are tainted by the other. The resulting model after employing molecular cutoffs, SPARE-AD2, assigned higher weights to regions including left hippocampus and less weights to regions including right middle temporal gyrus and right entorhinal area, compared with SPARE-AD1. SPARE-AD2 and SPARE-AD3 scores were generally more correlated with amyloid measures and similarly with measures of CSF total phosphorylated tau (pTau) and tau PET. Importantly, SPARE-AD2 and SPARE-AD3 were significantly less correlated with chronological age, which supported their increased specificity to Alzheimer’s disease-related brain changes. On the other hand, SPARE-BA2 Gap became less sensitive to differentiating Alzheimer’s disease versus cognitively normal individuals and less correlated with SPARE-AD2, supporting its increased specificity to age-related changes.

### Specific brain biomarkers of brain ageing and Alzheimer’s disease

In addition to using the molecular markers, our results also showed that adding individuals with Alzheimer’s disease to the training set of the brain ageing model can significantly reduce correlation between the two biomarkers. The resulting machine learning models learned brain patterns that were more specific to each of the two types of brain changes. For example, the SPARE-BA3 model avoided brain regions such as the inferior lateral ventricle, whose volume increase was associated not only with brain ageing in general, but also with the presence of Alzheimer’s disease. This was also evidenced by the progressively reduced relationship of the SPARE-BA scores (from SPARE-BA1 to SPARE-BA3) with Alzheimer’s disease-related molecular markers.

Conflating biomarkers of typical brain ageing and Alzheimer’s disease results in biased assessment of a person’s brain. The brain age gap has been associated with Alzheimer’s disease^4,5,15,16^; on the other hand, SPARE-AD has been reported to increase with age in cognitive normal individuals.^9,10^ Whether an individual with Alzheimer’s disease has an otherwise healthy brain or whether there is significant comorbid neurodegeneration related to other causes is important information for clinicians to estimate prognosis and to consider whether comorbidity-directed interventions may be worthwhile. Using the disentangled SPARE scores, one may be able to better differentiate between individuals with Alzheimer’s disease but otherwise healthy brain (top-left quadrant in the scatter plots in Fig. 3) and individuals with advanced brain ageing but no Alzheimer’s disease (bottom-right quadrant in the scatter plots in Fig. 3). This can potentially assist in determining the degree to which Alzheimer’s disease is driving neurodegeneration and the relative contribution of both processes to cognition and other outcomes in any individual. We may also find the degree of brain ageing in the setting of a particular severity of Alzheimer’s disease pathology influence the resilience to the pathology, thus informing clinical estimations of progression risk.

### Correlation with clinical variables

SPARE-AD2 and SPARE-AD3 were similarly or better correlated with amyloid and tau-related measurements, as well as with the number of APOE4 alleles, compared with SPARE-AD1. This suggests that the disentangled SPARE-AD, in addition to being less conflated with age, is still robustly linked to Alzheimer’s disease pathology. However, their correlations with psychometric test scores were somewhat reduced after this disentangling compared with SPARE-AD1. This is likely because these cognitive variables are not specific to the Alzheimer’s disease pathology alone, but also associated with the degree of background brain aging^59^ or other neurodegenerative processes which are often mixed with Alzheimer’s disease. Therefore, SPARE-AD1, which is sensitive to not only Alzheimer’s disease-related brain changes, but also to advanced brain ageing, likely better tracks with these mixed cognitive changes. Indeed, SPARE-AD1 is trained based on clinical status, which is closely linked to psychometric performance, whereas SPARE-AD2 and AD3 are trained based on molecular status regardless of the performance. Given these results, the disentangled SPARE-AD scores may not always be ideal if trying to track overall clinical status, especially where cognitive impairment reflects the effects of multiple pathologies, and different versions of SPARE scores may be used in combination to construct a more comprehensive description of an individual’s brain.

The correlations between SPARE-BA3 and Alzheimer’s disease-related clinical, molecular and genetic variables were consistently reduced and non-significant for the latter two, compared with SPARE-BA1 or SPARE-BA2. This emphasizes that the disentangled SPARE-BA scores are no longer influenced by brain changes associated specifically with Alzheimer’s disease, which would be useful for assessing the presence of accelerated brain ageing in individuals with Alzheimer’s disease without bias. Moreover, despite being disentangled, SPARE-BA3 Gap still significantly correlated with the psychometric measures, further supporting the notion that accelerated ageing itself is associated with declines in cognition, or, alternatively, that decelerated brain ageing is associated with resilience to age-associated cognitive decline. Thus, the combination of SPARE-BA3 with more selective SPARE-AD scores (SPARE-AD2 and SPARE-AD3), may best capture the contributions of both processes to cognitive decline, as evidenced by the complementary inclusion in nested multivariate models of cognition.

### Sample selection

The results underscore the importance of sample selection in building brain-based prediction models like the SPARE models. Inclusion and exclusion criteria used to define training samples significantly influenced neurodegenerative patterns learned by the models as well as the relationships of the derived scores to other variables. Therefore, caution is needed when the SPARE or other similar summary scores are used in novel populations or clinical settings to fully understand the implications of the scores based upon the methods applied to develop the underlying models. Here, amyloid status was primarily applied to select samples for the disentangled SPARE models, firstly, because of its high specificity to Alzheimer’s disease, but also because amyloid was the most commonly available molecular marker in the iSTAGING database. Tau status or the combination of imaging-based molecular markers specific to Alzheimer’s disease can further improve the model specificity.

As the goal was to achieve more disentangled, and therefore, more orthogonal axes, a direct construction of the two SPARE machine learning models with an added constraint of orthogonality and training them simultaneously could have been another solution, instead of indirectly redefining the training samples. However, not only would this require substantial modification to the current models, but it also poses several problems. First, because of the partial overlap of the affected regions, a trade-off between performance of a single metric and orthogonality is inevitable. By manipulating the training samples, guided by prior knowledge and hypotheses, we can avoid this statistical compromise and benefit from the full capacity of the machine learning models. The resulting stepwise improvements to the models are then relatively easier to interpret. The current solution is also much more versatile for future use, as opposed to requiring certain model structure. Moreover, enforcing orthogonality within the model would not test the hypothesis that the correlation between the two SPARE scores was indeed due to the loose diagnostic criteria and the overlapping affected brain regions associated with the two metrics. Taken together, we selected the current methods, and as a result, while more effort in the field is devoted to developing the best model structure and discovering the best hyperparameter settings, the current work underlines the importance of careful sample selection.

### Comorbidities of ageing

Ageing is a complex process that accompanies many comorbidities, including cancer, diabetes, cardiovascular disease and neurodegenerative disease.^1^ Differentiating between typical ageing and ageing-related disorders is difficult, if at all possible.^60,61^ The meaning of what is ‘typical’ and the degree to which a variety of age-associated processes contribute to brain integrity remain unclear. However, one may conceive that the current state of a human brain might be decomposed into the normative state corresponding to the chronological age plus weighted sum of abnormalities which further modulate brain structure. In this setting, the status of the brain can be described by a feature space where each axis corresponds to a type of an abnormality. While this is an oversimplification, as there are likely to be second order interactions and non-linearity of combined effects, developing such a simplified dimensional space may help researchers and clinicians by providing a snapshot of brain health.

Here, we introduced approaches to measure two such dimensions: advanced brain ageing and Alzheimer’s disease. There are many more axes to be added to this feature space, with each addition also potentially refining the normative population sample. For example, in the current analysis, the normal group (A−/CN) was defined as individuals without amyloid pathology. But it could, for example, further exclude those with diabetes from the control group and evaluate diabetes status distinctly as was done for Alzheimer’s disease here.^62,63^ A SPARE-diabetes score can be disentangled from SPARE-BA and SPARE-AD. As such, in the future, one brain scan may produce numerous such SPARE scores that are as much disentangled from each other, assessing the state of a human brain by measuring the presence and severity of neurodegeneration related to many pathologies.^64,65^

### Limitations and future work

There are a few limitations to the current work. First, there is variability in molecular measures across studies and techniques. Even within a single imaging technique, such as florbetapir PET, there exists a variety of methods for preparation and processing of the results, which causes significant between-study differences. Here, we circumvented the issue by linearly harmonizing the diagnostic cutoffs to match sensitivity and specificity, but the analyses linking the measures to the SPARE scores had to be performed in a study-wise manner.

Second, intuitively, a regression model is more suitable for developing biomarkers such as the SPARE scores than a classification model. This is because the separating plane in a classification model is most heavily governed by the borderline cases (especially with SVM). For example, in the SPARE-AD2 model, the separating plane was most likely defined by the more advanced cases of A−/CN and the least severe cases of Alzheimer’s disease Continuum in terms of the brain changes. This may explain the lower-than-expected classification accuracy of SPARE-AD2 at separating between the two groups and may have limited the correlation between SPARE-AD2 and Alzheimer’s disease-related molecular measurements. Here, a classification model was selected for SPARE-AD to be consistent with previous studies, but future work can explore regression model-based SPARE-AD scores.

Despite the limitations, the current work successfully demonstrated a decoupling of the SPARE-BA and SPARE-AD. Future work can explore tau status in combination with amyloid to define Alzheimer’s disease more carefully as more tau data become available. Here, we trained linear models using ROI volumes for better interpretability. Whether similar results yield using other structural or functional brain measures,^66,67^ or using more advanced, non-linear machine learning models can also be explored.^68,69^ Moreover, additional efforts to disentangle SPARE-BA from other ageing-related comorbidities will be valuable in delineating a complete set of most objective and specific biomarkers to evaluate an ageing brain.

## Supplementary Material

fcac117_Supplementary_Data
