# Supplementary material for: Disentangling Alzheimer’s disease neurodegeneration from typical brain ageing using machine learning
Source: Brain Commun. 2022 May 7;4(3):fcac117. doi: 10.1093/braincomms/fcac117 (PMC9123890; doi:10.1093/braincomms/fcac117)
Supplement: fcac117_Supplementary_Data [file fcac117_supplementary_data.docx]

# Supplementary Material for “Disentangling Alzheimer’s disease neurodegeneration from typical brain aging using machine learning”

**Supplementary** [**Table 1**](#Table_S2_ref)**. Summary of diagnostic criteria from pooled studies**

| **Study** | **Diagnosis** | **Criteria Summary** | **Reference** |
| --- | --- | --- | --- |
| ADNI | CN, MCI, AD | Memory complaint by patient or study partner  Abnormal memory function score on Logical Memory II subscale (delayed paragraph recall, paragraph A only) from the Wechsler Memory Scale – revised  **MCI:** Mini-Mental State Exam (MMSE) score between 24 and 30, CDR = 0.5, Memory Box score at least 0.5  **AD:** MMSE score between 20 and 26 (AD), CDR = 0.5 or 1.0, Memory Box score at least 1.0 | http://  adni.loni.usc.edu/  methods/  documents/ |
| BIOCARD | CN, MCI, AD | For individuals with a CDR > 0 and/or evidence of decline on cognitive testing, we followed the clinical diagnostic criteria in the National Institute on Aging/Alzheimer’s Association (NIA/AA) working group reports for the diagnosis of **MCI**^1^ and dementia due to **AD**^2^ | Albert et al.^1^  Sacktor et al.^3^ |
| BLSA | CN, MCI, AD | For individuals whose Blessed Information Memory Concentration score^4^ was 3 or greater,  CDR > 0,  or Dementia Questionnaire^5^ was abnormal,  **AD** diagnosis was based on Diagnostic and Statistical Manual of Mental Disorders, Revised Third Edition criteria,^6^ and  **MCI** diagnosis was based on the Mayo Clinic criteria.^7^ Accordingly, the diagnosis of MCI was given to subjects who had deficits limited to one or two areas of cognition (usually memory), with preservation of normal activities of daily living compared with other people of similar age. | O’Brien et al.^8^ |
| OASIS | CN, AD | The diagnosis was based on clinical information (derived primarily from a collateral source).  **AD**: CDR > 0 | Marcus et al.^9^ |

AD: Alzheimer’s disease, MCI: Mild Cognitive Impairment, CDR: Clinical Dementia Rating, CN: Cognitively Normal

**Supplementary** [**Methods 1**](#Table_S2_ref)**. Summary of Pooled Studies**

Data used in the preparation of this article were obtained in part from the Alzheimer’s Disease Neuroimaging Initiative (ADNI) database (adni.loni.usc.edu).^10,11^ The ADNI was launched in 2003 as a public-private partnership, led by Principal Investigator Michael W. Weiner, MD. The primary goal of ADNI has been to test whether serial MRI, PET, other biological markers, and clinical and neuropsychological assessment can be combined to measure the progression of mild cognitive impairment (MCI) and early Alzheimer’s disease. For up-to-date information, see www.adni-info.org.

The BIOCARD Study (officially entitled "Biomarkers of Cognitive Decline Among Normal Individuals: the BIOCARD cohort") is an extension of the Family Study/BIOCARD Study begun at National Institute of Mental Health (NIMH) in 1995.^12^ The overarching goal of the BIOCARD Study is to identify biomarkers associated with progression from normal cognitive status to cognitive impairment or dementia, with a particular focus on Alzheimer's disease. Investigators at the Johns Hopkins University School of Medicine began evaluating participants in 2009. The subjects are seen annually. At each visit there are assessments of medical and cognitive status, as well as acquisition of MRI, CSF, PET-PiB, and blood. The Johns Hopkins research team is the coordinating site of the Preclinical Alzheimer’s disease Consortium (PAC).

The Baltimore Longitudinal Study of Aging (BLSA) is an ongoing longitudinal study led by the National Institute of Aging (NIA).^13^ Established in Baltimore, Maryland, the USA in 1958, BLSA aims to characterize the process of aging in general, and healthy aging in particular. Beginning in 1986, BLSA introduced an extensive neuropsychological assessment to subjects older than 60 years. The neuroimaging sub-study of BLSA began in 1994, with annual or semi-annual MRI studies, cognitive testing, and clinical evaluations of a subset of 721 BLSA participants. For up-to-date information, see www.blsa.nih.gov.

The Open Access Series of Imaging Studies (OASIS) is a series of neuroimaging data sets that is publicly available for study and analysis.^9,14^ It is aimed at making neuroimaging datasets freely available to the scientific community and facilitating future discoveries in basic and clinical neuroscience. Previously released data for OASIS-Cross-sectional and OASIS-Longitudinal have been utilized for hypothesis driven data analyses, development of neuroanatomical atlases, and development of segmentation algorithms. OASIS-3 is a longitudinal neuroimaging, clinical, cognitive, and biomarker dataset for normal aging and Alzheimer’s disease. The OASIS datasets hosted by central.xnat.org provide the community with open access to a significant database of neuroimaging and processed imaging data across a broad demographic, cognitive, and genetic spectrum an easily accessible platform for use in neuroimaging, clinical, and cognitive research on normal aging and cognitive decline. For up-to-date information, see www.oasis-brains.org.

**Supplementary** [**Table 2**](#Table_S2_ref)**. 145 brain ROIs that were used as training features**

| 3rd ventricle | (R/L) Gyrus rectus |
| --- | --- |
| 4th ventricle | (R/L) Inferior occipital gyrus |
| (R/L) Accumbens area | (R/L) Inferior temporal gyrus |
| (R/L) Amygdala | (R/L) Lingual gyrus |
| Brain Stem | (R/L) Lateral orbital gyrus |
| (R/L) Caudate | (R/L) Middle cingulate gyrus |
| (R/L) Cerebellum exterior | (R/L) Medial frontal cortex |
| (R/L) Cerebellum (white matter) | (R/L) Middle frontal gyrus |
| (R/L) Hippocampus | (R/L) Middle occipital gyrus |
| (R/L) Inferior lateral ventricle | (R/L) Medial orbital gyrus |
| (R/L) Lateral ventricle | (R/L) Postcentral gyrus medial segment |
| (R/L) Pallidum | (R/L) Precentral gyrus medial segment |
| (R/L) Putamen | (R/L) Superior frontal gyrus medial segment |
| (R/L) Thalamus proper | (R/L) Middle temporal gyrus |
| (R/L) Ventral diencephalon | (R/L) Occipital pole |
| Cerebellar vermal lobules I-V | (R/L) Occipital fusiform gyrus |
| Cerebellar vermal lobules VI-VII | (R/L) Opercular part of inferior frontal gyrus |
| Cerebellar vermal lobules VIII-X | (R/L) Orbital part of inferior frontal gyrus |
| (R/L) Basal forebrain | (R/L) Posterior cingulate gyrus |
| (R/L) Frontal lobe (white matter) | (R/L) Precuneus |
| (R/L) Occipital lobe (white matter) | (R/L) Parahippocampal gyrus |
| (R/L) Parietal lobe (white matter) | (R/L) Posterior insula |
| (R/L) Temporal lobe (white matter) | (R/L) Parietal operculum |
| (R/L) Fornix | (R/L) Postcentral gyrus |
| (R/L) Anterior limb of internal capsule | (R/L) Posterior orbital gyrus |
| (R/L) Posterior limb of internal capsule including cerebral peduncle | (R/L) Planum polare |
| Corpus callosum | (R/L) Precentral gyrus |
| (R/L) Anterior cingulate gyrus | (R/L) Planum temporale |
| (R/L) Anterior insula | (R/L) Subcallosal area |
| (R/L) Anterior orbital gyrus | (R/L) Superior frontal gyrus |
| (R/L) Angular gyrus | (R/L) Supplementary motor cortex |
| (R/L) Calcarine cortex | (R/L) Supramarginal gyrus |
| (R/L) Central operculum | (R/L) Superior occipital gyrus |
| (R/L) Cuneus | (R/L) Superior parietal lobule |
| (R/L) Entorhinal area | (R/L) Superior temporal gyrus |
| (R/L) Frontal operculum | (R/L) Temporal pole |
| (R/L) Frontal pole | (R/L) Triangular part of the inferior frontal gyrus |
| (R/L) Fusiform gyrus | (R/L) Transverse temporal gyrus |

R/L: Right and Left hemisphere

**Supplementary Methods 2. Amyloid and tau cutoffs**

Conservative amyloid and tau cutoffs were selected to refine the training samples in the second and third versions of the SPARE models (SPARE-BA2, -BA3, -AD2, and -AD3). If there was a mismatch in acquisition dates between scans and the molecular measurements (52% of cases, average difference 69.3±64.3 days with the maximum of 365 days), the search span for date matching was gradually increased (±7, ±30, ±180, ±365 days) until there was a match. If more than one match was found within a range, the measurements were averaged. Most participants (3,071 out of 4,054) had data from multiple visits and multiple MRI scans (*n* = 15,533 total scans). The screening was performed in a way that would maximize the sample size for the machine learning model training.

CSF β-amyloid 1-42 (Aβ42) measures were provided by ADNI and BIOCARD, but, given differences in acquisition methods, there were significant study-wise discrepancies. First, for the ADNI participants, Aβ42<180pg/mL was labeled as amyloid positive (A+) and Aβ42>200pg/mL was labeled as amyloid negative (A-). Participants with Aβ42 between the two values were excluded from the training. Note that based on prior work, Aβ42<192pg/mL has been considered consistent with presence of cerebral amyloid using the Luminex platform.^15^ Then, the equivalent cutoffs for Aβ42 measurements from BIOCARD^16^ were calculated by matching two normal distribution fits: one for cognitively normal participants and another for individuals with either mild cognitive impairment or Alzheimer’s disease diagnosed clinically (MCI/AD). The equivalent cutoffs were 354pg/mL and 391pg/mL from comparing the distributions of the first groups, and 350pg/mL and 391pg/mL from comparing the distributions of the second groups. Then, the final cutoffs for BIOCARD were the average of the two sets (Aβ42<352pg/mL for A+ and Aβ42>391pg/mL for A-). Sensitivity (percentage of A+ in MCI/AD group) and specificity (percentage of A- in cognitively normal group) of Aβ42 were 70% and 55% for ADNI, and 61% and 57% for BIOCARD.

Pittsburgh compound B ([^11^C]PiB) standardized uptake value ratio (SUVR) measurements from amyloid-PET were provided by ADNI, BLSA, and OASIS, but there was study-wise discrepancy in measurements. First, for OASIS, [^11^C]PiB>1.50 (high amyloid burden group according to their previous study) was labeled as A+ and [^11^C]PiB<1.25 (low burden group) was labeled as A-.^17^ Using similar technique as with Aβ42 (only changing MCI/AD to only Alzheimer’s disease as OASIS did not have participants with mild cognitive impairment), the harmonized cutoffs for ADNI were 1.59 and 1.66 (a cutoff of 1.6 has been used in their previous study)^18^; for BLSA they were 1.01 and 1.09 (a cutoff of 1.064 has been used in their previous study).^19^ Sensitivity (percentage of A+ in Alzheimer’s disease group) and specificity of [^11^C]PiB were 79% and 50% for ADNI, 67% and 65% for BLSA, 73% and 74% for OASIS.

[^18^F]florbetapir SUVR measurements from amyloid-PET were provided by ADNI and OASIS and their distributions were similar in terms of the cutoffs. Previously, a cutoff of 1.11 was established.^20^ To be conservative, [^18^F]florbetapir>1.15 was labeled as A+ and [^18^F]florbetapir<1.05 was labeled as A- for both studies. Sensitivity (percentage of A+ in Alzheimer’s disease group) and specificity of [^18^F]florbetapir were 84% and 51% for ADNI, and 90% and 56% for OASIS. If more than one of the above three amyloid measurements were available per visit, all labels needed to agree for the final label.

Finally, CSF total tau measurements were provided by ADNI and BIOCARD and their distributions were similar in terms of the cutoffs. CSF total tau was only used to screen A+ cognitively normal participants to be included in the AD Continuum group. Previously, a cutoff of 93pg/mL was validated.^15^ To be conservative, CSF total tau>100pg/mL was labeled as tau positive (T+) for both studies. 17% of cognitively normal participants in ADNI and 12% of those in BIOCARD were labeled as T+.


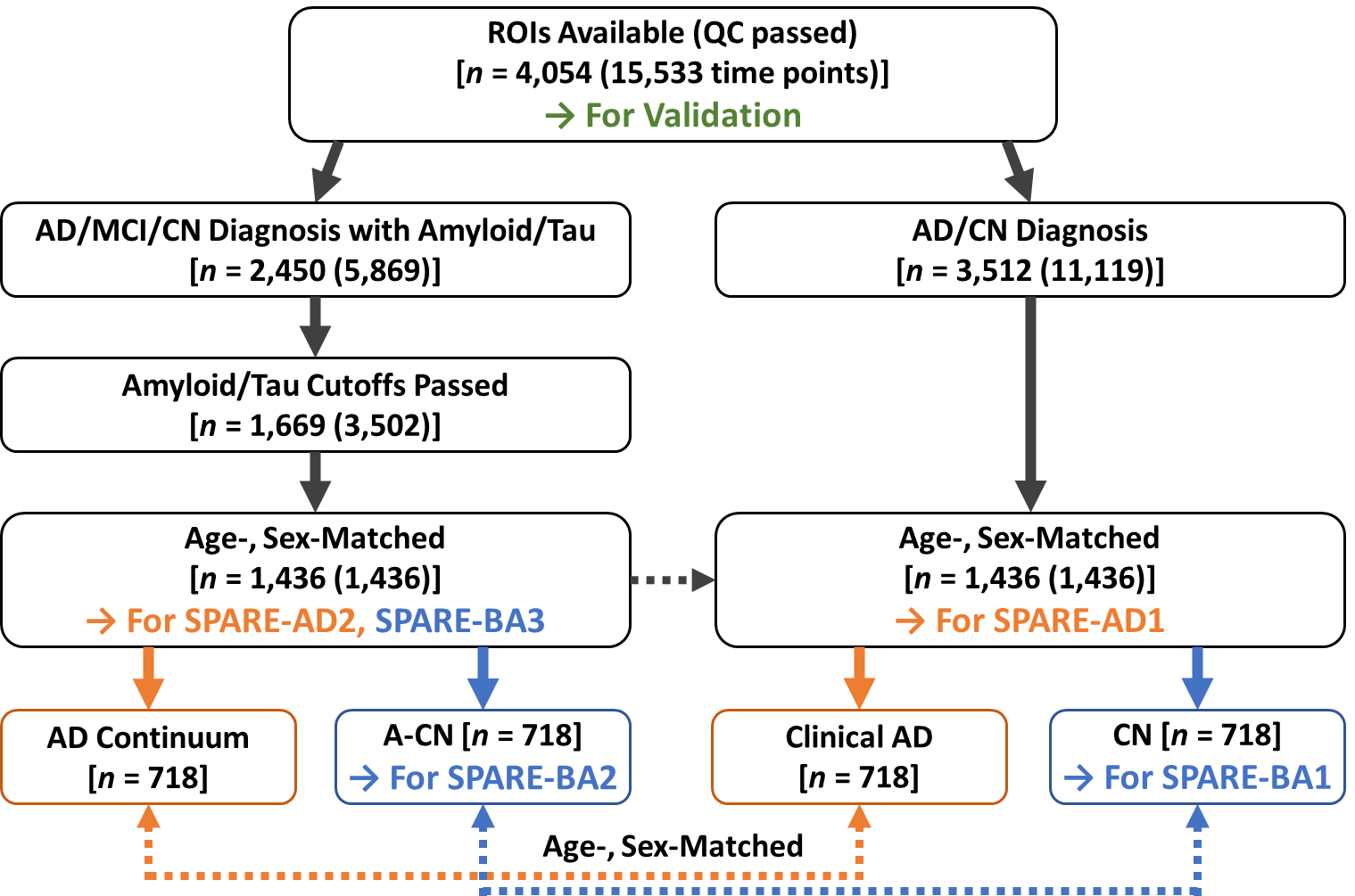


**Supplementary Figure 1** **A flowchart of the entire sample selection process.** Age- and sex-matched “AD Continuum” and “A-CN” groups were selected first, and then the “Clinical AD” and “CN” groups were selected to match between the four groups. *n* indicates the number of unique participants, while the numbers in parentheses indicate the numbers of total images. No two images from a participant entered a single model as training samples. AD = Alzheimer’s Disease; CN = Cognitively Normal; MCI = Mild Cognitive Impairment; QC = Quality Control

**Supplementary Methods 3. Machine learning model training**

All SPARE models were trained in MATLAB 2020a: “fitcsvm” function for SPARE-AD models and “fitrsvm” for SPARE-BA with linear kernels. Training samples were randomly split for 10-fold cross validation (CV). 145 MUSE ROIs that had been corrected for study- and sex-wise differences were normalized to z-scores per CV fold, based on the control group.

For SPARE-AD classification models, the misclassification cost (*C*) parameter was fine-tuned using a nested cross validation, while fixing all other hyperparameters at default values. The *C* parameter adjusts the amount the model is penalized for incorrectly classifying scans from either group, thereby affecting the sensitivity and specificity of the model. In our case, this would affect the distribution of the SPARE scores. The search span for *C* started with a coarse grid and was gradually tightened until the difference between sensitivity and specificity was within 1%. For the cross validation, the parameters varied. For the final models including all participants, *C* = [0,1;1.3,0] for SPARE-AD1 and *C* = [0,1;1.15,0] for SPARE-AD2 were selected.

For SPARE-BA regression models, the kernel scale and epsilon parameters were fine-tuned using a nested cross validation, while fixing all other hyperparameters at default values. The kernel scale, also known as the gamma parameter, affects the spacing between the data points. The epsilon parameter determines the margin of tolerance where no penalty is given to errors. For example, if a small epsilon is selected, the model is penalized for slight prediction errors. For the cross validation, the parameters varied. For the final models, the kernel scale of 6.1, 6.6, 6.3 and epsilon of 2.31, 1.14, 0.27 were selected for SPARE-BA1 to SPARE-BA3, respectively.

For the SPARE-BA regression models, a linear correction of predicted SPARE-BA scores was performed per fold to remove known systematic bias caused by regression dilution and regression towards the mean (old individuals predicted young, and vice versa).^21^

**Supplementary Table 3** **10-fold cross-validation results from the SPARE model training**

| **SVR**  **Results** | **MAE/RMSE/R^2^** | **SVM**  **Results** | **AUC /**  **Accuracy (%)** |
| --- | --- | --- | --- |
| SPARE-BA1 | 6.60/8.32/0.556 | SPARE-AD1 | 0.89 / 84.0 |
| SPARE-BA2 | 5.18/6.46/0.650 | SPARE-AD2 | 0.82 / 73.5 |
| SPARE-BA3 | 6.16/7.82/0.615 | SPARE-AD3^a^ | - |

^a^SPARE-AD3 was not an SVM model, but rather a simple model to residualize SPARE-AD2 with SPARE-BA3 scores.

AUC = Area-Under-the-Curve; MAE = Mean Absolute Error; RMSE = Root-Mean-Squared Error; SD = Standard Deviation.

****Supplementary Figure 2** **SPARE-BA test results.** Correlation between SPARE-BA and chronological age was computed on individuals whose images were not used in the training. The expected fit line is y = x. The linear fit is relatively similar in all three versions in cognitively normal (CN) individuals, while it is improved in individuals with either mild cognitive impairment (MCI) or Alzheimer’s disease (AD). CN = Cognitively Normal; MCI/AD = Mild Cognitive Impairment/Alzheimer’s Disease; MAE = Mean Absolute Error


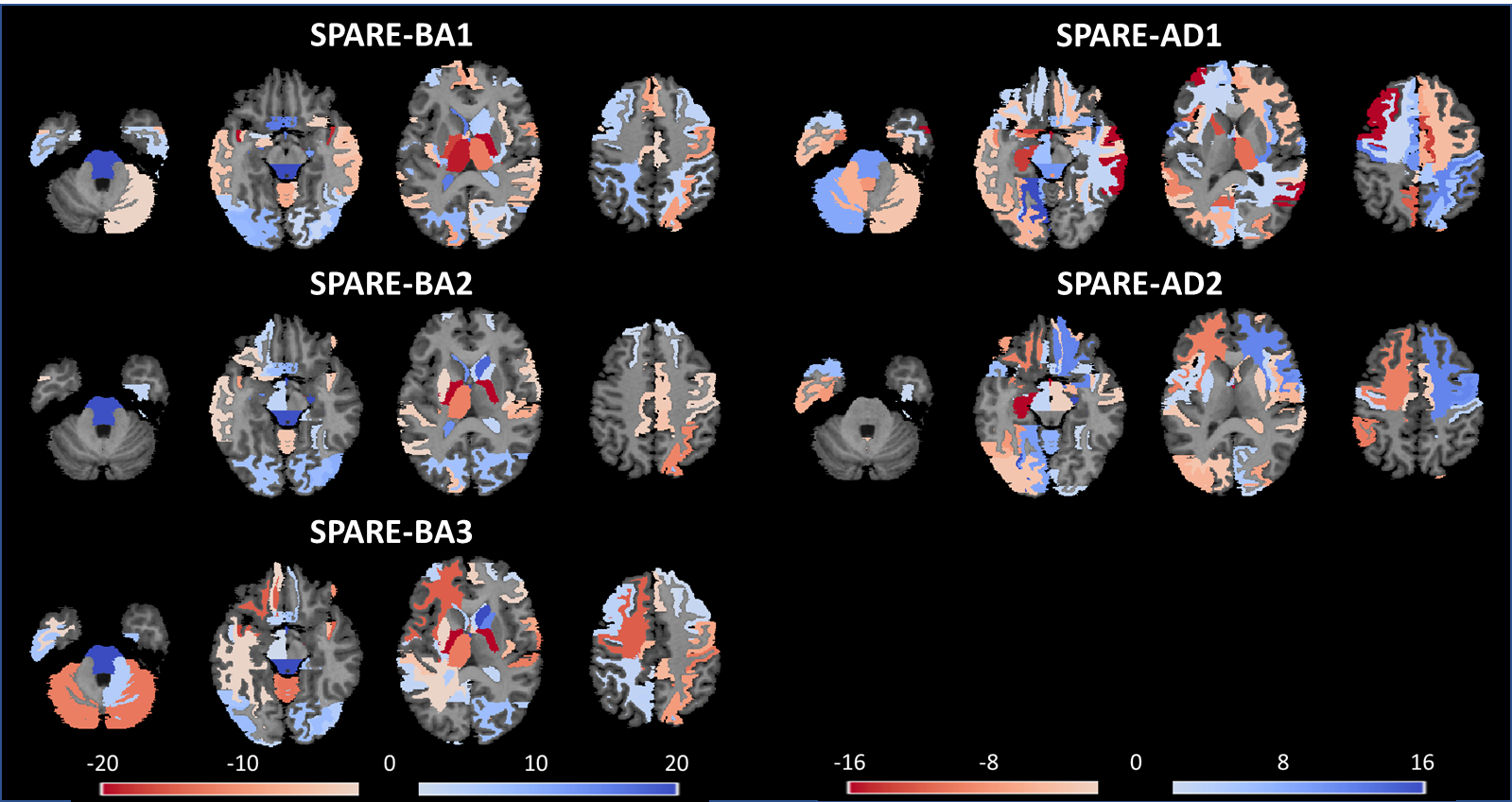
**Supplementary Figure 3** **Significance of Assigned Weights.** The color maps are based on the -log_10_ of the feature weights assigned by the final SPARE models. A negative value (red) indicates a negative weight, and a positive value (blue) indicates a positive weight. Note that because individual feature weights in a highly multivariate model are heavily influenced by interactions between the features, a negative weight may not necessarily correspond to a negative individual correlation. Therefore, interpretation of these maps should be performed in conjunction with Fig. 5 in the main text.

**Supplementary Table 4 ROIs with the most significant changes between SPARE models**

| **ROI Name** | **SPARE-BA** | | | | | | **SPARE-AD** | | | | |
| --- | --- | --- | --- | --- | --- | --- | --- | --- | --- | --- | --- |
|  | **BA1_ρ** | **BA2_ρ** | **BA3_ρ** | **BA1_w** | **BA2_w** | **BA3_w** | **AD1_ρ** | **AD2_ρ** | **AD3_ρ** | **AD1_w** | **AD2_w** |
| **More correlated with SPARE-BA** | |  |  |  |  |  |  |  |  |  |  |
| R Cerebellum Exterior | -0.36 | -0.32 | **-0.40*** | -1.46 (5) | -1.50 (4) | -3.09 (15) | -0.18 | **-0.03*** | **0.01*** | -0.30 (4) | 0.17 (1) |
| L Cerebellum Exterior | -0.37 | -0.33 | **-0.41*** | -1.12 (3) | -1.24 (3) | -3.00 (14) | -0.17 | **-0.04*** | 0.01 | 0.47 (8) | 0.14 (1) |
| R Pallidum | -0.41 | -0.40 | **-0.47*** | -1.27 (3) | -2.34 (7) | -3.82 (17) | -0.13 | **0.03**** | **0.09*** | -0.06 (1) | 0.15 (2) |
| Cerebellar Vermal Lobules I-V | -0.32 | -0.30 | **-0.36*** | -2.85 (10) | -2.53 (7) | -3.80 (15) | -0.15 | **0.00*** | 0.04 | -0.02 (0) | 0.18 (8) |
| R Frontal Lobe White Matter | -0.25 | -0.24 | **-0.31*** | -0.07 (0) | 0.31 (1) | -0.91 (2) | -0.08 | **0.04*** | 0.09 | -0.52 (4) | 0.87 (11) |
| L Frontal Lobe White Matter | -0.26 | -0.25 | **-0.32*** | -0.92 (3) | -0.48 (1) | -2.83 (17) | -0.08 | **0.03*** | 0.07 | 0.38 (3) | -0.81 (10) |
| R Anterior Limb of Internal Capsule | -0.51 | -0.50 | **-0.56*** | -3.56 (30) | -4.06 (31) | -5.34 (39) | -0.21 | **-0.06**** | **0.01*** | 0.14 (1) | 0.14 (1) |
| L Anterior Limb of Internal Capsule | -0.49 | -0.48 | **-0.55*** | -2.84 (18) | -3.66 (24) | -6.02 (45) | -0.20 | **-0.06*** | **0.00*** | 0.09 (1) | -0.04 (0) |
| **Less correlated with SPARE-AD only** | |  |  |  |  |  |  |  |  |  |  |
| Brain Stem | -0.35 | -0.33 | -0.36 | 4.19 (30) | 3.78 (21) | 6.52 (47) | -0.15 | **-0.02*** | 0.02 | 0.36 (10) | 0.14 (2) |
| R Thalamus Proper | -0.63 | -0.60 | -0.61 | -2.70 (14) | -1.43 (4) | -1.39 (3) | -0.40 | **-0.24**** | **-0.18*** | -0.52 (10) | -0.13 (1) |
| L Thalamus Proper | -0.63 | -0.61 | -0.62 | -3.16 (20) | -2.84 (14) | -3.20 (14) | -0.39 | **-0.24**** | **-0.17*** | 0.05 (0) | -0.18 (1) |
| R Medial Orbital Gyrus | -0.36 | -0.32 | -0.30 | 0.75 (1) | 1.14 (2) | 0.62 (1) | -0.25 | **-0.12*** | -0.08 | 0.14 (3) | 0.30 (11) |
| L Occipital Fusiform Gyrus | -0.35 | -0.35 | -0.32 | -1.03 (2) | -1.22 (2) | -2.03 (5) | -0.24 | **-0.10*** | -0.06 | 0.13 (4) | 0.34 (22) |
| R Precentral Gyrus | -0.54 | -0.53 | -0.55 | -2.70 (12) | -1.87 (5) | -3.13 (11) | -0.24 | **-0.09**** | **-0.03*** | -0.08 (1) | 0.35 (10) |
| L Precentral Gyrus | -0.48 | -0.50 | -0.50 | 1.22 (2) | -0.50 (1) | -1.40 (2) | -0.22 | **-0.10*** | **-0.04*** | 0.32 (12) | 0.16 (3) |
| L Posterior Limb of Internal Capsule | -0.32 | -0.31 | -0.32 | 1.28 (3) | -0.26 (0) | 0.93 (1) | -0.16 | **0.02**** | 0.06 | -0.44 (12) | 0.12 (1) |
| **Less correlated with SPARE-BA** | |  |  |  |  |  |  |  |  |  |  |
| L Hippocampus | -0.65 | -0.63 | **-0.53*** | -0.84 (1) | -1.46 (3) | -0.94 (1) | -0.72 | **-0.60**** | **-0.55*** | -0.51 (13) | -0.60 (17) |
| R Inferior Lateral Ventricle | 0.76 | **0.78*** | **0.64**** | 2.95 (14) | 5.36 (38) | 0.55 (1) | 0.56 | 0.54 | **0.48*** | 0.19 (3) | 0.49 (17) |
| L Inferior Lateral Ventricle | 0.77 | **0.79*** | **0.66**** | 3.38 (18) | 3.31 (16) | 1.14 (2) | 0.59 | **0.54*** | **0.48*** | 0.35 (9) | 0.21 (3) |
| L Frontal Operculum | -0.63 | -0.63 | **-0.58*** | -0.07 (0) | -1.82 (4) | 0.12 (0) | -0.42 | **-0.34*** | **-0.28*** | -0.25 (11) | -0.16 (4) |
| L Middle Frontal Gyrus | -0.51 | -0.50 | **-0.45*** | 2.15 (7) | 0.41 (0) | 2.44 (7) | -0.37 | **-0.24*** | **-0.19*** | -0.43 (16) | -0.12 (1) |
| R Middle Temporal Gyrus | -0.58 | -0.55 | **-0.44*** | -2.35 (8) | -1.46 (3) | -0.50 (0) | -0.55 | **-0.36**** | -0.32 | -0.44 (17) | 0.00 (0) |
| L Precuneus | -0.50 | -0.50 | **-0.41*** | -0.59 (1) | -0.27 (0) | 2.43 (7) | -0.36 | **-0.24*** | -0.20 | -0.38 (11) | -0.12 (1) |
| L Parietal Operculum | -0.47 | -0.48 | **-0.44*** | -0.07 (0) | 1.20 (2) | 1.27 (2) | -0.26 | **-0.13*** | **-0.08*** | 0.15 (4) | 0.38 (21) |
| R Planum Polare | -0.68 | **-0.63*** | -0.62 | -3.86 (20) | -2.54 (7) | -3.98 (16) | -0.36 | **-0.26*** | **-0.20*** | 0.27 (11) | 0.12 (2) |
| L Planum Polare | -0.69 | **-0.63*** | -0.62 | -5.69 (43) | -3.08 (11) | -4.21 (18) | -0.37 | **-0.28*** | **-0.22*** | 0.05 (1) | 0.00 (0) |

MUSE ROIs with both significant changes in correlation with the SPARE scores (*P_FDR_* < 1E-10) and significant weights in any of the models (-log_10_(*P_FDR_*) > 10, shown in parentheses) are displayed.

**P_FDR_* < 0.05; ***P_FDR_* < 1E-10; L = left hemisphere; R = right hemisphere.

**

**Supplementary Figure 4** **Brain regions most and least affected by Alzheimer’s disease.** The left two columns show brain regions that are most different between AD Continuum and A-/CN while most correlated with age in A-/CN (*P_FDR_* < 1.7E-17). The right two columns show brain regions that are least different between the two groups (*P* > 0.3) while most correlated with age in A-/CN (*P_FDR_* < 1.0E-15). Odd columns show their brain volume plotted against chronological age. Even columns show the significance of the feature weights. Compared to SPARE-BA2, the SPARE-BA3 model consistently assigned more significant weights to brain regions on the right, as the regions on the left became significantly less correlated with age after the addition of AD Continuum group to the A-/CN group (*P* =0.012, paired *t*-test).

**Supplementary Table 5** **Spearman correlations between SPARE scores and Alzheimer’s disease-related variables**

| **Variable** | **Study** | **Group** | ***n*** | **AGE** | **SPARE-BA1** | **SPARE-BA2** | **SPARE-BA3** | **SPARE-BA1 Gap** | **SPARE-BA2 Gap** | **SPARE-BA3 Gap** | **SPARE-AD1** | **SPARE-AD2** | **SPARE-AD3** |
| --- | --- | --- | --- | --- | --- | --- | --- | --- | --- | --- | --- | --- | --- |
| **Molecular (amyloid)** |  |  |  |  |  |  |  |  |  |  |  |  |  |
| CSF Aβ42 | ADNI | ALL | 1055 | -0.126** | -0.300** | -0.302** | -0.129**† | -0.277** | -0.267** | -0.065*† | -0.431** | **-0.441**** | -0.433** |
|  | ADNI | CN | 282 | -0.220* | -0.131* | -0.152* | -0.142* | 0.014 | 0.023 | -0.007 | -0.072 | -0.106 | -0.084 |
|  | ADNI | MCI+AD | 773 | -0.095* | -0.248** | -0.250** | -0.076*† | -0.243** | -0.231** | -0.017† | -0.387** | -0.400** | -0.399** |
| [^18^F]Florbetapir (AV45) SUVR | ADNI | ALL | 1091 | 0.079* | 0.235** | 0.233** | 0.089*† | 0.239** | 0.224** | 0.050† | 0.396** | **0.401**** | 0.399** |
|  | ADNI | CN | 332 | 0.009 | -0.042 | -0.019 | -0.044 | -0.06 | -0.045 | -0.064 | 0.114* | 0.140* | 0.139* |
|  | ADNI | MCI+AD | 759 | 0.101* | 0.255** | 0.248** | 0.100*† | 0.249** | 0.222** | 0.038† | 0.384** | 0.396** | 0.396** |
| [^11^C]PiB SUVR | OASIS | ALL | 484 | **0.332**** | 0.310** | 0.301** | 0.264** | 0.047 | -0.049 | -0.03 | 0.209** | 0.199** | 0.166* |
|  | OASIS | CN | 436 | 0.273** | 0.193** | 0.186* | 0.176* | -0.063 | -0.147* | -0.088 | 0.067 | 0.069 | 0.04 |
|  | ADNI | MCI+AD | 83 | -0.11 | 0.008 | -0.015 | -0.058 | 0.162 | 0.107 | 0.086 | 0.230* | 0.187 | 0.197 |
| **Molecular (tau)** |  |  |  |  |  |  |  |  |  |  |  |  |  |
| CSF Total Tau | ADNI | ALL | 1041 | 0.160** | 0.278** | 0.266** | 0.109*† | 0.218** | 0.190** | -0.004† | **0.431**** | 0.416** | 0.410** |
|  | ADNI | CN | 281 | 0.237** | 0.119 | 0.148* | 0.12 | -0.059 | -0.052 | -0.039 | 0.011 | 0.076 | 0.063 |
|  | ADNI | MCI+AD | 760 | 0.132* | 0.239** | 0.219** | 0.064† | 0.194** | 0.161** | -0.049† | 0.425** | 0.397** | 0.397** |
| CSF Total Phosphorylated Tau | ADNI | ALL | 1054 | 0.056 | 0.146** | 0.138** | -0.000† | 0.132** | 0.120* | -0.042† | 0.312** | 0.321** | **0.321**** |
|  | ADNI | CN | 281 | 0.154* | -0.034 | -0.024 | -0.021 | -0.190* | -0.207* | -0.158* | -0.009 | 0.047 | 0.049 |
|  | ADNI | MCI+AD | 773 | 0.018 | 0.116* | 0.105* | -0.040† | 0.137* | 0.128* | -0.059† | 0.302** | 0.308** | 0.313** |
| Tau PET  (entorhinal area) | ADNI | ALL | 635 | 0.104* | 0.228** | 0.199** | 0.107* | 0.196** | 0.128* | 0.050 | 0.377** | 0.285** | 0.274** |
|  | ADNI | CN | 377 | 0.054 | 0.038 | 0.020 | 0.017 | -0.006 | -0.029 | -0.017 | 0.132* | 0.032 | 0.028 |
|  | ADNI | MCI+AD | 258 | 0.008 | 0.234* | 0.191* | 0.031† | 0.294** | 0.201* | 0.033† | **0.477**** | 0.430** | 0.429** |
| Tau PET  (inferior temporal gyrus) | ADNI | ALL | 635 | 0.051 | 0.188** | 0.161* | 0.077 | 0.206** | 0.148* | 0.077 | 0.290** | 0.273** | 0.267** |
|  | ADNI | CN | 377 | 0.055 | 0.054 | 0.041 | 0.038 | 0.033 | 0.016 | 0.028 | 0.093 | 0.084 | 0.078 |
|  | ADNI | MCI+AD | 258 | -0.069 | 0.184* | 0.141* | -0.014 | 0.314** | 0.232* | 0.062† | 0.402** | 0.410** | **0.415**** |
| **Psychometric** |  |  |  |  |  |  |  |  |  |  |  |  |  |
| MMSE | ADNI | ALL | 1764 | -0.170** | -0.505** | -0.492** | -0.281**† | -0.499** | -0.461** | -0.226**† | **-0.628**** | -0.585**† | -0.567** |
|  | BLSA | CN | 849 | -0.196** | -0.188** | -0.199** | -0.211** | -0.048 | 0.007 | -0.068 | -0.073* | -0.076* | -0.049 |
|  | ADNI | MCI+AD | 1310 | -0.184** | -0.503** | -0.487** | -0.269**† | -0.486** | -0.439** | -0.193**† | -0.609** | -0.557**† | -0.540** |
| MOCA | ADNI | ALL | 1194 | -0.236** | -0.492** | -0.487** | -0.306**† | -0.456** | -0.414** | -0.197**† | **-0.581**** | -0.534** | -0.511** |
|  | ADNI | CN | 358 | -0.340** | -0.292** | -0.309** | -0.277** | -0.107 | -0.067 | -0.066 | -0.208* | -0.137* | -0.102 |
|  | ADNI | MCI+AD | 836 | -0.229** | -0.493** | -0.477** | -0.286**† | -0.459** | -0.399** | -0.159**† | -0.566** | -0.518** | -0.497** |
| ADAS-COG-11 | ADNI | ALL | 1760 | 0.183** | 0.528** | 0.515** | 0.282**† | 0.522** | 0.482** | 0.213**† | **0.677**** | 0.640** | 0.621** |
|  | ADNI | CN | 453 | 0.165* | 0.130* | 0.141* | 0.114* | 0.051 | 0.055 | 0.009 | 0.130* | 0.185* | 0.173* |
|  | ADNI | MCI+AD | 1307 | 0.186** | 0.501** | 0.484** | 0.247**† | 0.487** | 0.438** | 0.156**† | 0.652** | 0.610** | 0.594** |
| ADAS-COG-13 | ADNI | ALL | 1759 | 0.187** | 0.545** | 0.536** | 0.299**† | 0.535** | 0.499** | 0.229**† | **0.702**** | 0.659**† | 0.639** |
|  | ADNI | CN | 453 | 0.198** | 0.220** | 0.234** | 0.194** | 0.134* | 0.137* | 0.078 | 0.184* | 0.218** | 0.193** |
|  | ADNI | MCI+AD | 1306 | 0.185** | 0.512** | 0.499** | 0.259**† | 0.496** | 0.450** | 0.172**† | 0.668** | 0.624** | 0.607** |
| Logical Memory  (immediate) | ADNI | ALL | 1681 | -0.092* | -0.473** | -0.455** | -0.230**† | -0.514** | -0.480** | -0.237**† | **-0.641**** | -0.586**† | -0.572** |
|  | ADNI | CN | 437 | -0.031 | -0.092 | -0.107* | -0.084 | -0.09 | -0.110* | -0.088 | -0.089 | -0.102* | -0.092 |
|  | ADNI | MCI+AD | 1244 | -0.096* | -0.436** | -0.412** | -0.188**† | -0.468** | -0.421** | -0.173**† | -0.594** | -0.536**† | -0.525** |
| Logical Memory  (delayed) | ADNI | ALL | 1681 | -0.102** | -0.462** | -0.447** | -0.239**† | -0.495** | -0.469** | -0.234**† | **-0.652**** | -0.605**† | -0.590** |
|  | ADNI | CN | 437 | -0.034 | -0.066 | -0.085 | -0.07 | -0.044 | -0.074 | -0.057 | -0.081 | -0.097 | -0.087 |
|  | ADNI | MCI+AD | 1244 | -0.124** | -0.422** | -0.402** | -0.214**† | -0.431** | -0.391** | -0.170**† | -0.594** | -0.546** | -0.533** |
| Trail Making Test  (part A) | ADNI | ALL | 1627 | 0.216** | 0.438** | **0.439**** | 0.283**† | 0.381** | 0.353** | 0.182**† | 0.427** | 0.394** | 0.373** |
|  | BLSA | CN | 896 | 0.436** | 0.386** | 0.387** | 0.383** | 0.024 | -0.146**† | -0.046† | 0.166** | 0.106* | 0.051 |
|  | ADNI | MCI+AD | 1215 | 0.172** | 0.401** | 0.403** | 0.243**† | 0.368** | 0.342** | 0.155**† | 0.368** | 0.348** | 0.330** |
| Trail Making Test  (part B) | ADNI | ALL | 1608 | 0.206** | 0.473** | 0.463** | 0.281**† | 0.434** | 0.396** | 0.188**† | **0.519**** | 0.484** | 0.463** |
|  | BLSA | CN | 900 | 0.398** | 0.362** | 0.367** | 0.365** | 0.044 | -0.108*† | -0.009† | 0.186** | 0.101* | 0.049 |
|  | ADNI | MCI+AD | 1195 | 0.187** | 0.439** | 0.426** | 0.247**† | 0.414** | 0.370** | 0.151**† | 0.461** | 0.436** | 0.418** |
| **Genetic** |  |  |  |  |  |  |  |  |  |  |  |  |  |
| APOE4 Alleles | ADNI | ALL | 1753 | -0.121** | 0.102** | 0.095* | -0.031† | 0.212** | 0.215** | 0.080*† | 0.296** | 0.293** | **0.300**** |
|  | BLSA | CN | 831 | -0.137* | -0.123* | -0.127* | -0.125* | -0.006 | 0.037 | 0.013 | -0.011 | -0.036 | -0.015 |
|  | ADNI | MCI+AD | 1301 | -0.142** | 0.054 | 0.047 | -0.069*† | 0.177** | 0.176** | 0.054† | 0.261** | 0.250** | 0.259** |

Highest correlations per variable are highlighted.

*Corrected *P* < 0.05; **Corrected *P* < 0.0001

†Significant difference from the value on the left (*P* < 0.05).

**Supplementary Table 6** **Multivariate linear regression models using orthogonalized SPARE scores to predict clinical variables**

| **Response Variable (*y*)** | ***n*** | **SPARE-BA3** | **SPARE-BA3 Gap** | **SPARE-AD3** | **Likelihood Ratio *(p)^22^***† |
| --- | --- | --- | --- | --- | --- |
| MMSE | 1310 | -0.094* | -0.109* | -0.501** |  |
|  |  | -0.166** |  | -0.494** | 0.0018 |
|  |  |  | -0.170** | -0.512** | 0.0048 |
| MOCA | 836 | -0.135* | -0.068 | -0.465** |  |
|  |  | -0.181** |  | -0.458** | **0.0882** |
|  |  |  | -0.156** | -0.485** | 0.0018 |
| ADAS-Cog 11 | 1307 | 0.097* | 0.081* | 0.537** |  |
|  |  | 0.150** |  | 0.532** | 0.0111 |
|  |  |  | 0.143** | 0.548** | 0.0028 |
| ADAS-Cog 13 | 1306 | 0.111* | 0.084* | 0.564** |  |
|  |  | 0.165** |  | 0.559** | 0.0069 |
|  |  |  | 0.155** | 0.576** | 0.0018 |
| Logical Memory (immediate) | 1244 | -0.077* | -0.111* | -0.480** |  |
|  |  | -0.151** |  | -0.475** | 0.0018 |
|  |  |  | -0.161** | -0.488** | 0.0227 |
| Logical Memory (delayed) | 1244 | -0.110* | -0.068* | -0.472** |  |
|  |  | -0.155** |  | -0.469** | 0.0425 |
|  |  |  | -0.139** | -0.484** | 0.0018 |
| Trail Making Test (part A) | 1215 | 0.07 | 0.094* | 0.293** |  |
|  |  | 0.132** |  | 0.289** | 0.0135 |
|  |  |  | 0.140** | 0.299** | **0.0638** |
| Trail Making Test (part B) | 1195 | 0.120* | 0.053 | 0.381** |  |
|  |  | 0.154** |  | 0.378** | **0.1255** |
|  |  |  | 0.130** | 0.393** | 0.0018 |

Only individuals with mild cognitive impairment (MCI) or Alzheimer’s disease (AD) were included in the models.

y ~ SPARE-BA3 + SPARE-BA3 Gap + SPARE-AD3 for unrestricted models (first row in each y). Blank cells indicate dropped variables.

**P_FDR_* < 0.05; ***P_FDR_* < 0.0001

†Significance indicates that the restricted model fit is significantly worse than the unrestricted model (*P_FDR_* > 0.05 are highlighted)

**Further Acknowledgements**

Data used in the preparation of this article were in part obtained from the Alzheimer's Disease Neuroimaging Initiative (ADNI) database. As such, the investigators within the ADNI contributed to the design and implementation of ADNI and provided data but did not participate in analysis or writing of this report. For a complete list of investigators involved in ADNI see: http://www.loni.ucla.edu/ADNI/Data/ADNI_Authorship_List.pdf.

ADNI is funded by the National Institute on Aging, the National Institute of Biomedical Imaging and Bioengineering, and through generous contributions from the following: Abbott, AstraZeneca AB, Bayer Schering Pharma AG, Bristol-Myers Squibb, Eisai Global Clinical Development, Elan Corporation, Genentech, GE Healthcare, GlaxoSmithKline, Innogenetics, Johnson and Johnson, Eli Lilly and Co., Medpace, Inc., Merck and Co., Inc., Novartis AG, Pfizer Inc, F. Hoffman-La Roche, Schering-Plough, Synarc, Inc., and Wyeth, as well as nonprofit partners the Alzheimer's Association and Alzheimer's Drug Discovery Foundation, with participation from the US Food and Drug Administration. Private sector contributions to ADNI are facilitated by the Foundation for the National Institutes of Health (www.fnih.org). The grantee organization is the Northern California Institute for Research and Education, and the study is coordinated by the Alzheimer's Disease Cooperative Study at the University of California, San Diego. ADNI data are disseminated by the Laboratory for Neuro Imaging at the University of California, Los Angeles. This research was also supported by NIH grants P30 AG010129, K01 AG030514, and the Dana Foundation. The National Cell Repository for Alzheimer's Disease (NIH grant U24 AG021886) provided support for DNA and cell line banking and processing for ADNI.

**References**

1. Albert M, Soldan A, Gottesman R, et al. Cognitive changes preceding clinical symptom onset of mild cognitive impairment and relationship to ApoE genotype. *Curr Alzheimer Res.* 2014;11(8):773-784.

2. McKhann GM, Knopman DS, Chertkow H, et al. The diagnosis of dementia due to Alzheimer's disease: recommendations from the National Institute on Aging-Alzheimer's Association workgroups on diagnostic guidelines for Alzheimer's disease. *Alzheimers Dement.* 2011;7(3):263-269.

3. Sacktor N, Soldan A, Grega M, et al. The BIOCARD Index: A Summary Measure to Predict Onset of Mild Cognitive Impairment. *Alzheimer Dis Assoc Disord.* 2017;31(2):114-119.

4. Blessed G, Tomlinson BE, Roth M. The association between quantitative measures of dementia and of senile change in the cerebral grey matter of elderly subjects. *Br J Psychiatry.* 1968;114(512):797-811.

5. Silverman JM, Breitner JC, Mohs RC, Davis KL. Reliability of the family history method in genetic studies of Alzheimer's disease and related dementias. *Am J Psychiatry.* 1986;143(10):1279-1282.

6. Association AP. *Diagnostic and Statistical Manual of Mental Disorders.* Vol 3. Revised (DSM-II-R) ed. Washington D.C.: American Psychiatric Press, Inc; 1987.

7. Petersen RC. Mild cognitive impairment as a diagnostic entity. *J Intern Med.* 2004;256(3):183-194.

8. O'Brien RJ, Resnick SM, Zonderman AB, et al. Neuropathologic studies of the Baltimore Longitudinal Study of Aging (BLSA). *J Alzheimers Dis.* 2009;18(3):665-675.

9. Marcus DS, Wang TH, Parker J, Csernansky JG, Morris JC, Buckner RL. Open Access Series of Imaging Studies (OASIS): cross-sectional MRI data in young, middle aged, nondemented, and demented older adults. *J Cogn Neurosci.* 2007;19(9):1498-1507.

10. Mueller SG, Weiner MW, Thal LJ, et al. The Alzheimer's disease neuroimaging initiative. *Neuroimaging Clin N Am.* 2005;15(4):869-877, xi-xii.

11. Jack CR, Jr., Bernstein MA, Fox NC, et al. The Alzheimer's Disease Neuroimaging Initiative (ADNI): MRI methods. *J Magn Reson Imaging.* 2008;27(4):685-691.

12. Greenwood PM, Lambert C, Sunderland T, Parasuraman R. Effects of apolipoprotein E genotype on spatial attention, working memory, and their interaction in healthy, middle-aged adults: results From the National Institute of Mental Health's BIOCARD study. *Neuropsychology.* 2005;19(2):199-211.

13. Stone JL, Norris AH. Activities and attitudes of participants in the Baltimore longitudinal study. *J Gerontol.* 1966;21(4):575-580.

14. LaMontagne PJ, Benzinger TL, Morris JC, et al. OASIS-3: Longitudinal Neuroimaging, Clinical, and Cognitive Dataset for Normal Aging and Alzheimer Disease. *medRxiv.* 2019.

15. Shaw LM, Vanderstichele H, Knapik-Czajka M, et al. Cerebrospinal fluid biomarker signature in Alzheimer's disease neuroimaging initiative subjects. *Ann Neurol.* 2009;65(4):403-413.

16. Moghekar A, Li S, Lu Y, et al. CSF biomarker changes precede symptom onset of mild cognitive impairment. *Neurology.* 2013;81(20):1753-1758.

17. Lopes Alves I, Heeman F, Collij LE, et al. Strategies to reduce sample sizes in Alzheimer’s disease primary and secondary prevention trials using longitudinal amyloid PET imaging. *Alzheimer's Research & Therapy.* 2021;13(1):82.

18. Ewers M, Insel P, Jagust WJ, et al. CSF biomarker and PIB-PET-derived beta-amyloid signature predicts metabolic, gray matter, and cognitive changes in nondemented subjects. *Cereb Cortex.* 2012;22(9):1993-2004.

19. Kamil RJ, Bilgel M, Wong DF, Resnick SM, Agrawal Y. Vestibular Function and Beta-Amyloid Deposition in the Baltimore Longitudinal Study of Aging. *Front Aging Neurosci.* 2018;10:408.

20. Landau SM, Mintun MA, Joshi AD, et al. Amyloid deposition, hypometabolism, and longitudinal cognitive decline. *Ann Neurol.* 2012;72(4):578-586.

21. Smith SM, Vidaurre D, Alfaro-Almagro F, Nichols TE, Miller KL. Estimation of brain age delta from brain imaging. *Neuroimage.* 2019;200:528-539.

22. Lewis F, Butler A, Gilbert L. A unified approach to model selection using the likelihood ratio test. *Methods in Ecology and Evolution.* 2011;2(2):155-162.
